# Structural Differences between the Genomes of *Deinococcus radiodurans* Strains from Different Laboratories

**DOI:** 10.3390/genes15070847

**Published:** 2024-06-27

**Authors:** Ksenija Zahradka, Davor Zahradka, Jelena Repar

**Affiliations:** Division of Molecular Biology, Ruđer Bošković Institute, 10000 Zagreb, Croatia; kvlahovi@irb.hr (K.Z.); zahradka@irb.hr (D.Z.)

**Keywords:** structural variation, genome rearrangements, mobile elements, IS, SNR, alternative end-joining

## Abstract

The bacterium *Deinococcus radiodurans* is known to efficiently and accurately reassemble its genome after hundreds of DNA double-strand breaks (DSBs). Only at very large amounts of radiation-induced DSBs is this accuracy affected in the wild-type *D. radiodurans*, causing rearrangements in its genome structure. However, changes in its genome structure may also be possible during the propagation and storage of cell cultures. We investigate this possibility by listing structural differences between three completely sequenced genomes of *D. radiodurans* strains with a recent common ancestor—the type strain stored and sequenced in two different laboratories (of the ATCC 13939 lineage) and the first sequenced strain historically used as the reference (ATCC BAA-816). We detected a number of structural differences and found the most likely mechanisms behind them: (i) transposition/copy number change in mobile interspersed repeats—insertion sequences and small non-coding repeats, (ii) variable number of monomers within tandem repeats, (iii) deletions between long direct DNA repeats, and (iv) deletions between short (4–10 bp) direct DNA repeats. The most surprising finding was the deletions between short repeats because it indicates the utilization of a less accurate DSB repair mechanism in conditions in which a more accurate one should be both available and preferred. The detected structural differences, as well as SNPs and short indels, while being important footprints of deinococcal DNA metabolism and repair, are also a valuable resource for researchers using these *D. radiodurans* strains.

## 1. Introduction

The bacterium *Deinococcus radiodurans* can survive and accurately repair hundreds of simultaneous DNA double-strand breaks (DSBs) caused by γ-radiation, desiccation, or other DNA damaging agents [1,2,3,4]. For this reason, *D. radiodurans* has long been the subject of DSB repair studies. Despite the potent and accurate repair, mistakes do seem to happen at extreme levels of DSBs, implying inaccurate DNA repair at very high levels of DSBs [5,6].

DSB repair based on RecA-dependent homologous DNA matching is highly accurate but can cause genome structural changes in the presence of interspersed repetitive sequences by homologously matching ectopic repetitive sequences [7,8]. *D. radiodurans* contains a high number of interspersed repetitive sequences—mobile genetic elements, which consist of both insertion sequences (ISs with a length of ~500 to ~3000 bp, encoding transposases) and small non-coding repeats (SNRs with a length of 60–215 bp) [9,10]. However, the presence of repeats does not seem to notably hinder the ability of γ-irradiated *D. radiodurans* to accurately repair hundreds of simultaneous DSBs.

The efficient and accurate repair of DSBs in *D. radiodurans*, which is not hindered by the presence of repetitive sequences, is considered a possible adaptation to DSB-causing dry environments such as deserts [2,3,11]. It follows that *D. radiodurans* would hypothetically produce fewer mistakes than, for example, *Escherichia coli*, under equivalent DSB-inducing conditions even though the two species contain a comparable number of interspersed repetitive sequences [12].

The main mechanism of DSB repair in *D. radiodurans*, i.e., the extended-synthesis-dependent strand annealing (ESDSA), is dependent on homologous DNA matching but is also atypical for a bacterium. ESDSA relies on the existence of multiple genome copies within the same cell, i.e., on the existence of an unbroken homologous DNA stretch overlapping each DSB; this unbroken stretch of DNA is then used as a template for massive DNA synthesis [13]. The accuracy of the process is strongly dependent on the (long) homology between DNA strands (template- and DSB site-strand), which leads to the extension and attachment of DNA strands after DSBs. Classical homologous recombination (HR) has also been detected in *D. radiodurans* as a final stage of DSB repair during which the ESDSA-produced long linear intermediates are matured into unit-size circular chromosomes [13].

Alternatively, more inaccurate mechanisms have been proposed to act as backups in DSB repair in *D. radiodurans*: single-strand annealing (SSA) [14,15,16] and alternative end-joining (A-EJ) [17]. They utilize shorter homologies for strand reattachment (>25 bp and 2–20 bp, respectively) and are, therefore, more prone to mistakes and the production of genome structural change. Different mechanisms can usually be distinguished by the features of a resulting structural change, features such as the length of homologies at the ends of deleted DNA, or the linear distance between misattached DNA ends [18,19,20,21]. These less accurate mechanisms of DSB repair are thought to only be employed as a backup in case of an overwhelming number of DSBs. 

Spontaneous DSBs, i.e., DSBs not caused by external agents, can also appear in bacteria, although not many at once, during normal growth and DNA replication [22,23,24]. It follows that genome structural change can also spontaneously arise in normally growing bacterial populations. In fact, spontaneous genome change in laboratory strains has been previously detected for several bacteria, including *Vibrio cholerae* and *E. coli* [25,26]. The frequency of a genome change in the population can be increased by selection or drift. Interestingly, selection has been known to act in the genome of *E. coli* under specific laboratory conditions [25,27]. However, how laboratory storage and propagation will influence the generation and frequency of genome change in a laboratory strain population is often unclear; here, we characterize such genome changes in different strains of *D. radiodurans*.

In this paper, we analyze differences between the genomes of *D. radiodurans* strains descended from the first *D. radiodurans* isolate obtained in 1956 [28]. The samples of this isolate have been separated at an unknown point in time and stored and propagated in different laboratories, and their genomes were subsequently completely sequenced and published independently by different groups. We use the published genome sequences resulting from this “accidental” diversification experiment to ask: Has the efficient and accurate repair of DSBs in *D. radiodurans* protected its genome structure from rearrangements during divergence in different laboratories? 

Our starting hypothesis was that there would be no traces of inaccurate repair of spontaneous DSBs in these recently diverged *D. radiodurans* strains due to the high accuracy of the main DSB repair mechanism. More precisely, there would be no genome rearrangements (deletions, duplications, inversions, or translocations) bordered (and, presumably, caused) by repeated sequences that may have caused the derailment of the DSB repair mechanism. To test this hypothesis, we specifically studied structural genome changes (i.e., changes affecting regions of DNA of approximately 1 kb or longer) because they carry potential implications for DSB repair in this bacterium. We detect, characterize, and classify structural changes and make conclusions about the mechanisms by which they came about. We discuss the detected mechanisms in the context of the expected extreme accuracy of DSB repair in *D. radiodurans*. 

Additionally, we characterize the single nucleotide polymorphisms (SNPs) and short insertions/deletions (indels) and identify affected genes. We expect that the comprehensive list of differences between the deinococcal genomes will benefit researchers studying specific genes in the investigated *D. radiodurans* strains.

## 2. Materials and Methods

The genome of *D. radiodurans* is multipartite. The sequences used in this paper are available in GenBank under IDs (i) AE000513.1, AE001825.1, AE001826.1, and AE001827.1 (chromosome I, chromosome II, megaplasmid MP1, and plasmid CP1, respectively, from the first completely sequenced genome of *D. radiodurans* [29] of the strain ATCC BAA-816) and (ii) CP038663.1, CP038664.1, CP038665.1, and CP038666.1 (chromosome I, chromosome II, megaplasmid MP1, and plasmid CP1, respectively, of the ATCC 13939 lineage, obtained in 2002 from the laboratory of Suzanne Sommer (Université Paris-Sud, Orsay, France), subsequently stored at −80 °C in 15% glycerol and PacBio sequenced and assembled by our laboratory [17]). The first genome is referred to as genome-1999 throughout the paper, and the second one is genome-2021, referring to the years in which they have been published. The listed sequences for genome-1999 and genome-2021 are available in the NCBI Assembly database under IDs ASM856v1 and ASM2054668v1 and have been evaluated internally by NCBI to have completeness scores of 97.17% and 98.67%, respectively. Additionally, we have included a third published and completely sequenced *D. radiodurans* genome in our analysis [30], the genome elements of which are available in the GenBank under IDs CP015081.1, CP015082.1, CP015083.1, and CP015084.1. Its NCBI genome assembly ID is ASM163882v1 and is marked to have a completeness score of 98.81%. This genome will be referred to as genome-2016, referring to the year in which it was published. To simplify the analysis and alignment to the other two genomes, genome-2016 was fully circled (i.e., the very long bordering DNA overlaps present in some genome elements were cut), and the genome reverse complement was calculated for the genome elements oriented differently than the corresponding elements in the other two genomes.

The phylogenetic distance between the different *D. radiodurans* strains was estimated using chromosome I of each genome and by calculating their distance using the andi software v.0.12 [31]. Based on the distances, a neighbor-joining phylogenetic tree was constructed using MEGA v. 11.0.13 [32]. The *Deinococcus deserti* chromosome I that we used is available under GenBank ID CP001114.1.

Differences between the genomes were detected using the methodology described in our previous paper [17]. Briefly, the genomes were aligned and compared using the program DNAdiff v.1.3. from the Mummer package [33]. DNAdiff reports both structural differences and SNPs between pairs of strains. Based on this report of structural differences, the sequences encompassing the nucleotides reported as affected by the difference, i.e., +/−100 bp, were extracted from the genomes and used for structural change characterization to identify footprints of possible mechanisms that caused the change. Blastn was used to identify structural changes stemming from the transpositional activity; the affected genome regions were matched to the ISFinder database of IS sequences using the blastn option of ISFinder (available as an online tool accessed during April of 2024) [34] and to the previously characterized SNR (small non-coding repeats) sequences [9] using blastn (BLAST v2.9.0+, [35]) locally. The results were used to detect which of these changes corresponded to a movement of an insertion sequence or SNR sequence. The sequences pinpointed by DNAdiff were also examined by Tandem Repeats Finder (TRF) [36] as well as Inverted Repeats Finder (IRF) [37], online tools accessed in April 2024. The structural changes that could not be explained by the transposition of repetitive sequences or variation in the number of tandem repeats were examined manually through the blastn alignment [35] to the corresponding change from the other *D. radiodurans* strain or to itself.

The SNP output of DNAdiff was mapped by a custom Java script to the GenBank annotations of genes in genome-1999 in order to examine the affected genes, i.e., genes whose sequence differed between the two genomes.

## 3. Results

**Figure 1:**
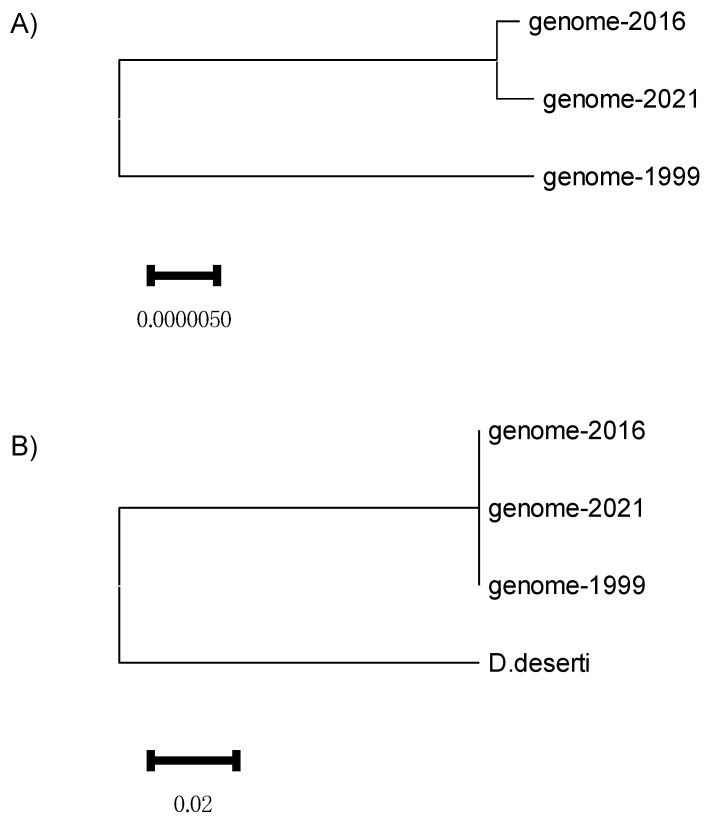
Phylogenies computed from the complete chromosome I based on the distance d_a_ ([31], see Methods) between the (**A**) *Deinococcus radiodurans* genomes used in this paper and (**B**) the same genomes with the *Deinococcus deserti* genome as an outgroup.

We compared the genomes of three *D. radiodurans* strains that have diverged during processes of growth and storage in different laboratories. These genomes will be referred to as genome-1999, genome-2016, and genome-2021 according to the year in which they were published (see Section 2). The phylogenetic tree shows that genome-2021 and genome-2016 are very closely related and genome-1999 is more distant (Figure 1A). However, these are very small distances, which become unnoticeable when these genomes are compared to a closely related outgroup such as *Deinococcus deserti* (Figure 1B). This is expected since the three investigated strains of *D. radiodurans* originate from the same recent (1956) isolate. Throughout Section 3 and Section 4, if genome-2016 is not specifically mentioned, it means that its structure corresponds to the structure of genome-2021, as would be expected from their phylogenetic closeness (Figure 1).

**Figure 2:**
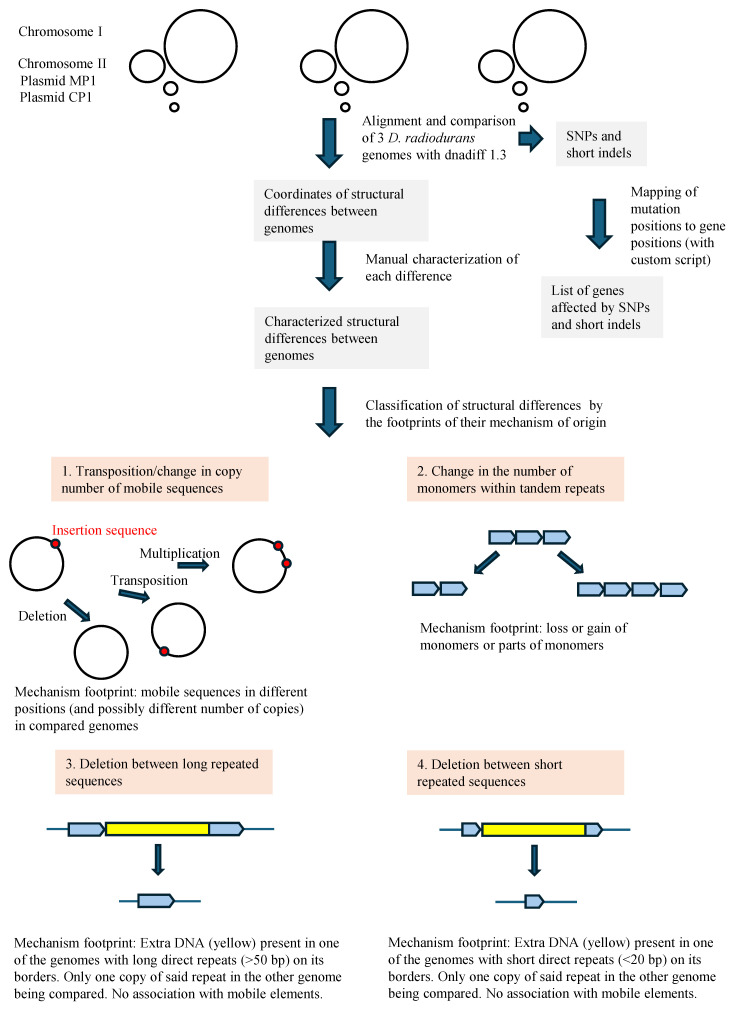
Workflow of our analysis, with graphical representations of detected types of structural genome change in *D. radiodurans*.

A comparison of the genomes of three *D. radiodurans* strains revealed changes in the genome structure that we characterized and classified into four types according to their probable mechanism of origin (Figure 2).

First, the strains differ in the transposition/copy number of mobile interspersed repetitive sequences (Table 1 and Table 2). We detected the movement of two types of mobile interspersed repetitive sequences in the genome of *D. radiodurans*: insertion sequences (ISs) and small non-coding repeats (SNRs). Most of these were detected when comparing genome-1999 and genome-2021 (Table 1). In total, there were only two additional structural changes when comparing genome-2021 to genome-2016, i.e., two additional transpositions (Table 2). Of the 12 families of ISs previously found in genome-1999 [9,10], we detected positional changes of five, namely, ISDra2, ISDra3, ISDra5, ISDra6, and IS2621 (Table 1 and Table 2). All five of these ISs have been previously shown to be transpositionally active [38,39]. Some of these IS changes do not include a variation in the copy number—e.g., one copy of ISDra3 is found in a different position in genome-1999 when compared to the genome-2021 (Table 1). However, some of the changes do include a variation in the copy number—e.g., there are six more copies of ISDra2 in genome-1999 than in genome-2021 (Table 1). In such cases, it is unclear whether ISs were deleted in one or have multiplied in the other genome. Of the nine types of SNRs previously found in genome-1999 [9,10], we detected an additional copy of SNR4 when comparing it to genome-2021, demonstrating a copy number change and potential mobility of SNR4 (Table 1).

The second type of change in the genome structure that we detected is a variable number of monomers within tandem repeats (Table 3). Generally, tandem repeats consist of different numbers of adjacent copies of a monomer sequence. The multiplication/deletion of monomers probably occurs by misalignments during replication [37], but given the length of monomers in Table 3, deletions of monomers could also conceivably occur through the SSA mechanism of DSB repair or A-EJ.

The third type of change in the genome structure that we detected is deletions between long interspersed direct repeats, in which one copy of the repeat was also deleted (Table 4). The probable mechanism of origin for these events is ESDSA or classical DSB repair via homologous recombination. However, the possibility of local misassembly in the genome-1999 sequence should also be considered (see Section 4).

The fourth type of change in genome structure that we detected is deletions between short (<20 bp) interspersed direct repeats, in which one copy of the repeat was also deleted (Table 5). The distinction between long and short DNA repeats was made on the basis of the implicated mechanism responsible for the structural change. We were specifically interested in repeats <20 bp as they imply the alternative end-joining (A-EJ) mechanism of DSB repair. 

We also detected SNPs and short indels when comparing the three genomes. We mapped the SNPs and indels to the genes annotated in genome-1999 to create a resource for researchers studying specific genes in either one of the *D. radiodurans* strains. We report the full list of genes in Table S1 (for a comparison of genome-1999 vs. genome-2021) and Table S2 (for a comparison of genome-1999 vs. genome-2016), and the summary of annotations of affected genes is given in Table 6. We also included several proteins of general interest in Table 6. A frameshift SNP in the SSB gene (Table 6), as well as one in the neighboring intergenic sequence (Table S1), was previously reported as a misassembly in genome-1999 [40].

## 4. Discussion

The detection of deletions that have occurred during the divergence of *D. radiodurans* strains in different laboratories is a surprising finding in a bacterium thought to be extremely DSB-repair proficient. This finding contradicts our hypothesis that we would not find any traces of inaccurate DSB repair in these closely related strains.

Moreover, the deletions happen not only between long repetitive sequences but also between short ones (4–10 bp). Previously, deletions between short (4–11 bp) repeated sequences have only been shown after the inactivation of more accurate, RecA-dependent, and long homology-based mechanisms of DSB repair (within the genome-2021 strain [6,17]). However, our present results suggest that such deletions might occur even after spontaneous DSBs in fully repaired proficient cells. This is surprising because deletions between short repeated sequences imply the activity of a less accurate mechanism of DSB repair (A-EJ or SSA, with the shortness of homology implying A-EJ [17,18,21]) in conditions in which a more accurate mechanism (e.g., ESDSA or HR) should be both available and preferred. Indeed, strains used in this research are highly radiation-resistant, showing high capacity for accurate DSB repair.

We can envision the following explanations for the surprising use of a less accurate DSB repair mechanism after spontaneous DSBs: (i) some laboratory storage conditions may be conducive to DNA damage and other forms of stress (e.g., storage in stabs [25]) and/or (ii) a less accurate DSB repair mechanism may be a significant part of the *D. radiodurans* toolbox for genome repair and maintenance (e.g., see [14,41]).

The transposition/multiplication of ISs is common in bacteria [42] and, therefore, less unexpected, even in *D. radiodurans*. Most of the differently positioned ISs that we detected were inserted into chromosome I (14/15), consistent with the previous observation that chromosome I has the most ISs per bp [9,10]. Notably, a higher copy number of ISDra2 in genome-1999 (Table 1) is possibly due to the expansion of ISDra2, which can be induced by DNA damage [38]. Alternatively, some of the copies might have been deleted in genome-2021.

The first event in Table 4 is a special case of deletion associated with an IS sequence: one transposed sequence of ISDra2 in genome-1999 replaces a ~6 kb sequence present in genome-2021. This deletion is likely a consequence of the ISDra2 transposition; IS elements are known to sometimes cause genome instability [42]. ISDra2 transposes to the right of the target sequence 5-TTGAT-3 [43], which is also the case here. The host sequence on the other side of the transposed ISDra2 varies widely [43], indicating that there are no special sequence requirements for the attachment of the right end of the IS sequence. As this IS sequence requires DNA to be single-stranded during transposition [44], it is easy to imagine how secondary or tertiary structures of single-stranded host DNA might cause the IS to jump over and thus delete some host DNA. However, it is also possible that two ISDra2 transpositions occurred on either side of the ~6 kb deletion fragment and that the deletion between them was mediated by homologous recombination. Interestingly, a similar event was reported previously in *Mycobacterium tuberculosum* [45]. Other than this event, no other deletion was associated with an IS or SNR mobile sequence.

Surprisingly, the length of the deleted DNA in either of the compared genomes varied by up to 6065 bp, and many deletions encompassed coding sequences, including several metabolic genes (see Table 4 and Table 5). The effect of the deletion of these genes in the laboratory conditions is unclear. We speculate that it might slightly speed up the replication; the streamlining of bacterial genomes through deletions is expected as an effect of selection pressure for faster growth [46]. Interestingly, the inactivation of some metabolic genes and subsequent accumulation of metabolic intermediates might hypothetically confer higher resistance to γ radiation/desiccation, which happened in *E. coli* subjected to selection pressure for higher radiation resistance [47]. However, it is also possible that the inactivation of a few genes does not significantly influence doubling time and/or radiation survival in the rich medium typically used for *D. radiodurans* growth and experiments. The lack of functional analysis is the main limitation of the present study as we predominantly focus on structural differences between the genomes. The impact of observed genome differences on function remains to be elucidated in future studies.

When investigating differences between phylogenetically close genome sequences such as the ones presented in this paper, it should be considered whether identified genome differences that involve long repeated sequences might be due to the misassembly of reads during the genome sequencing process. Ironically, the misassembly at long repeated sequences will closely resemble genuine cell events of recombination between ectopic repetitive sequences. Therefore, the third event in Table 3, as well as events 2–4 in Table 4, should be approached with caution. They all involve long repeats in genome-1999, which was sequenced and assembled from reads that were on average much shorter [29] than the PacBio reads used for genome-2021 [17]. Nonetheless, we do not expect the deletion events between short repeats to be due to misassembly as the average length of reads used for the assembly of genome-1999 greatly surpasses the length of repeats in Table 5.

The results of this work showed that *D. radiodurans* strains of nominally the same genotype can hide many genetic differences, including genomic rearrangements. These differences could have arisen over decades due to different ways of storing the same strains in several laboratories around the world. Strains are now stored frozen at −70 °C or −80 °C as a standard procedure. This form of storage ensures both the long-term viability of the strains and the stability of their genomes. In 1956, when *D. radiodurans* was first isolated, this was not the standard. Presumably, agar stab cultures or freezing at −20 °C was predominately used at that time. Storage at −20 °C is not ideal because the cells lose their viability after a certain time, which then requires more frequent replenishment of stocks and regrowth of strains. It is possible that more frequent cycles of regrowth and freezing open space for spontaneous genomic changes. On the other hand, storage in stabs enables a long lifespan in the stationary phase, which is not an inert state as once thought, but can be the source of various changes in the genome [48]. Therefore, researchers in the field of bacterial genetics and genomics must be constantly aware of the dynamism of the bacterial genome and the possibility that genomic changes can take place at any moment, even in seemingly non-stressful laboratory conditions.

## 5. Conclusions

Notably, some types of genome structural changes between the three investigated genomes were more expected (transposition/multiplication of ISs and change in length of tandem repeats), and some were unexpected in a bacterium as proficient in DSB repair as *D. radiodurans* (long deletions, especially between short repeated sequences) and indicate the use of a less accurate DSB repair mechanism in conditions in which a more accurate one should be both available and preferred.

Our results demonstrate that presuming isogeneity between commonly shared bacterial strains is not always safe and investigating differences between them can give us new information. Information on common structural changes and their mechanisms of origin in the genomes of bacteria propagated in the laboratory will help navigate further research in the field of structural change and genome rearrangements, especially in the models of DSB repair such as *D. radiodurans*. In addition to being important in terms of inferences about deinococcal DNA metabolism and repair, the detected genome changes are a valuable resource for the researchers using the investigated *D. radiodurans* strains.

## Figures and Tables

**Table 1:** List of mobile interspersed repetitive sequences—insertion sequences (ISs) and small non-coding repeats (SNRs) differently positioned when comparing genome-1999 and genome-2021. The list is ordered by position on the genome element. Extra DNA means DNA (in this case, a copy of IS or SNR) present in one genome but not the other.

IS/ SNR	The Investigated Genome in Which Extra DNA Is Present	Extra DNA Starts at bp:	Genome Element	Extra DNA Overlaps an Annotated Gene at Its Edge (Location and GenBank Annotation *); Genes Are Numbered for Clarity as There Are Instances of Multiple Genes Being Affected.
ISDra3	genome-1999	252969	Chromosome I	[i]<1..>1070,23S ribosomal RNA, DR_r02
ISDra2/ IS8301	genome-1999	674926	Chromosome I	None
ISDra6	genome-2021	694904	Chromosome I	[i]<1..16, E5E91_03580, hypothetical protein
IS2621	genome-1999	881456	Chromosome I	[i]<1..31, hypothetical protein, DR_0869
SNR4	genome-2021	969707	Chromosome I	None
ISDra2/ IS8301 **	genome-1999	994752	Chromosome I	[i]<1..70, DR_0977, AAF10554.1, phosphoenolpyruvate carboxykinase, [ii]1705..>1743, DR_0980, AAF10557.1, glutamate dehydrogenase, putative, loss of 6 kb DNA
IS2621	genome-1999	1337894	Chromosome I	None
ISDra2/ IS8301	genome-1999	1387636	Chromosome I	[i]<1..71, DR_1380, AAF10959.1, hypothetical protein
ISDra2/ IS8301	genome-1999	1611598	Chromosome I	[i]complement(1675..>1745), DR_1594, AAF11161.1, hypothetical protein
IS2621	genome-1999	1638129	Chromosome I	[i]complement(1302..>1322), DR_1619, conserved hypothetical protein
ISDra3	genome-2021	1745642	Chromosome I	[i]complement(<1), E5E91_08855, incomplete, partial on complete genome, missing N-terminus, DUF4238 domain-containing protein
ISDra2/ IS8301	genome-1999	1953199	Chromosome I	[i]complement(<1..38), DR_1931, conserved hypothetical protein, [ii]complement(1673..>1742), DR 1934, hypothetical protein
ISDra2/ IS8301	genome-1999	2321426	Chromosome I	[i] complement(<1..38), DR_2322, serine protease, subtilase family, C-terminal fragment, [ii]complement(1673..>1742), DR_2325, serine protease, subtilase family, N-terminal fragment
IS2621	genome-2021	374859	Chromosome II	[i] <1..3, E5E91_15400, incomplete, partial on the complete genome, missing C-terminus, tetratricopeptide repeat protein

* The GenBank annotation includes the position of the affected gene in a specific GenBank format, which is best understood through examples, e.g., 1302..>1322. In this example, a region with a length of 1322 bp is investigated. Within the region, there is a gene with a starting position at nucleotide 1302, and the gene extends through (“..”) and beyond (“>”) the last nucleotide of the investigated region, which is at position 1322. ** The appearance of this IS in the new position is correlated with a long (>6 kb) deletion in the same position.

**Table 2:** List of mobile interspersed repetitive sequences—insertion sequences (ISs) differently positioned when comparing genome-2016 and genome-2021. The list is ordered by the position on the genome element. Extra DNA means DNA (in this case, a copy of IS) present in one genome but not the other.

IS	The Investigated Genome in Which Extra DNA Is Present	Extra DNA Starts at bp:	Genome Element	Extra DNA Overlaps an Annotated Gene at Its Edge
ISDra5	genome-2016	656554	Chromosome I	None
IS2621	genome-2016	1753516	Chromosome I	None

**Table 3:** Features of detected differences in tandem repeats between the two genomes and their statistics, reported by Tandem Repeats Finder [36].

Monomer Size	Copy Number in Genome-1999	Starting Position (bp) in Genome-1999	Copy Number in Genome-2021	Starting Position (bp) in Genome-2021	Percent Matches, Percent Indels	Genome Element
24	3.1	1118441	5.1	1119952	100,0	Chromosome I
21	1	2457333	3.9	2453220	79,9	Chromosome I
218	2	45488	1	1	97,0	Plasmid CP1

**Table 4:** Features of deletions associated with long repetitive sequences.

Deletion—Length of Unique Sequence Present in Only One Genome (bp)	Genome with Unique Non-Deleted DNA	Genome Element	The Position (bp) at Which Unique Non-Deleted DNA Starts	Repetitive Sequence at Deletion Boundaries (L and R)	Unique Non-Deleted DNA Present in Only One of the Two Investigated Genomes Contains Genes (Location and GenBank Annotations *). Genes Are Numbered for Clarity as There Are Instances of Multiple Genes Being Affected.
6065	genome-2021	Chromosome I	991922	Special case: extra DNA exchanged with one IS sequence (IS2621) (see Section 4)	[i]<1..276, E5E91_05075, phosphoenolpyruvate carboxykinase, [ii] 492..1343, E5E91_05080, hypothetical protein, [iii] 1734..2879, pdhA, E5E91_05085, pyruvate dehydrogenase (acetyl-transferring) E1 component subunit α, [iv] 3132..4157, E5E91_05090, α-ketoacid dehydrogenase subunit β, [v] 4404..5663, E5E91..05095, Glu/Leu/Phe/Val dehydrogenase, [vi] 5728..>6065, E5E91_05100, Glu/Leu/Phe/Val dehydrogenase
24	genome-1999	Chromosome I	1960529	526 bp	[i]complement(<1..>24), DR_1939, putative; polyphosphate kinase, authentic frameshift
917	genome-1999	Chromosome II	288082	600 bp	[i]complement(<1..331), DR_A0268, adenine deaminase-related protein, [ii]complement(524..880), DR_A0269, hypothetical protein
48	genome-1999	Megaplasmid	96697	59 bp	[i]complement(32..>48), DR_B0078, this region contains authentic frameshift

* The GenBank annotation includes the position of the affected gene in a specific GenBank format, which is best understood through examples, e.g., 1302..>1322. In this example, a region with a length of 1322 bp is investigated. Within the region, there is a gene with a starting position at nucleotide 1302, and the gene extends through (“..”) and beyond (“>”) the last nucleotide of the investigated region, which is at position 1322.

**Table 5:** Features of deletions associated with short repetitive sequences.

Deletion—Length of Unique Sequence (bp)	Unique Non-Deleted DNA in Strain	Genome Element Identity	The Position (bp) at Which Unique Non-Deleted DNA Starts	Repetitive Sequence at Deletion Boundaries (L and R)	Unique Non-Deleted DNA Present in Only One of the Two Investigated Genomes Contains Genes (Location and GenBank Annotations *). Genes Are Numbered for Clarity as There Are Instances of Multiple Genes Being Affected.
1900	genome-1999	Chromosome I	1230616	CGGC	[i]288..1043, E5E91_06355, “regulator”, [ii]1040..>1900, E5E91_06360, EamA family transporter
147	genome-1999	Chromosome I	1234925	CAGGCGGCGC	[i]<1..>147, E5E91..06375, glycosyltransferase
1178	genome-1999	Chromosome I	1819776	ACCCAGCGGG	[i]74..922, E5E91_09235, hypothetical protein

* The GenBank annotation includes the position of the affected gene in a specific GenBank format which is best understood through examples, e.g., 1302..>1322. In this example, a region with a length of 1322 bp is investigated. Within the region, there is a gene with a starting position at nucleotide 1302, and the gene extends through (“..”) and beyond (“>”) the last nucleotide of the investigated region, which is at position 1322.

**Table 6:** Summary of annotations of genes affected by SNPs and short indels between the two investigated *D. radiodurans* strains. The reported SNPs and indels have been mapped to the gene annotations in genome-1999 and summarized. Separate from the summarized categories of proteins, we included 7 proteins of general interest also affected by detected SNPs/indels (DNA topoisomerase I, FtsE, elongation factor TU, DNA polymerase III (β subunit), FtsK, SSB, and SbcC).

Annotation of the Gene with the SNP/Indel	Number of Positions Affected by SNP/Indel in Genome-1999 Compared to Genome-2021	Number of Positions Affected by SNP/Indel in Genome-1999 Compared to Genome-2016
Hypothetical protein	147	190
Conserved hypothetical protein	33	42
Transposase (putative)	49	49
DNA-binding response regulator	3	2
Intergenic SNPs	189	240
DNA topoisomerase I	1	1
FtsE	3	3
Elongation factor TU	1	0
DNA polymerase III, β subunit	1	1
Cell division protein FtsK (putative)	5	5
Single-stranded DNA-binding protein	1	1
Exonuclease SbcC	11	11
Other	114	135
Total	558	680

## Data Availability

The original contributions presented in the study are included in the article/supplementary material, further inquiries can be directed to the corresponding author.

## Supplementary Materials

The following supporting information can be downloaded at: https://www.mdpi.com/article/10.3390/genes15070847/s1, Table S1: Annotations of genes affected by SNPs and indels between two *D. radiodurans* strains (genome-1999 was used as the reference DNA sequence for this comparison, and genome-2021 was used as the query). Table S2: Annotations of genes affected by SNPs and indels between two *D. radiodurans* strains (genome-1999 was used as the reference DNA sequence for this comparison, and genome-2016 was used as the query).
