# Supplementary material for: Structural Differences between the Genomes of Deinococcus radiodurans Strains from Different Laboratories
Source: Genes (Basel). 2024 Jun 27;15(7):847. doi: 10.3390/genes15070847 (PMC11276467; doi:10.3390/genes15070847)
Supplement: Supplementary file 1 [file genes-15-00847-s001.zip › genes-3050677-supplementary.pdf]

Structural Differences between the Genomes of *Deinococcus radiodurans* Strains from Different Laboratories

Ksenija Zahradka, Davor Zahradka and Jelena Repar *

Division of Molecular Biology, Ruđer Bošković Institute, 10000 Zagreb, Croatia

*Correspondence: jelena.repar@irb.hr

Supplementary Table S1. Annotations of genes affected by SNPs and indels between two *D. radiodurans* strains – genome-1999 (see Methods for details) was used as the reference DNA sequence for this comparison, and genome-2021 as the query.

Coordinates of the gene with the SNP	Function of the gene with the SNP	Position of the SNP in the reference	Character in the reference	Character in the query	Reference GenBank ID
complement(1..1182)	DNA polymerase III, beta subunit	145	G	.	AE000513.1
-	-	1618	A	.	AE000513.1
37446..38756	serine hydroxymethyl transferase	37941	A	G	AE000513.1
complement(52646..54421)	serine/threonine protein kinase, putative	53823	T	C	AE000513.1
-	-	54429	C	.	AE000513.1
55152..55580	hypothetical protein	55517	G	.	AE000513.1
-	-	65577	C	G	AE000513.1
-	-	65578	G	C	AE000513.1
complement(66759..68792)	peptidyl-prolyl cis-trans isomerse, putative	66949	.	C	AE000513.1
100371..100802	single-stranded	100733	.	G	AE000513.1

	DNA-binding protein				
-	-	100960	.	G	AE000513.1
114105..114995	hypothetical protein	114697	.	G	AE000513.1
complement(116822..117385)	acetyl-CoA carboxylase, bitoin carboxyl carrie	117214	A	G	AE000513.1
complement(116822..117385)	acetyl-CoA carboxylase, bitoin carboxyl carrie	117250	T	.	AE000513.1
complement(116822..117385)	acetyl-CoA carboxylase, bitoin carboxyl carrie	117282	.	C	AE000513.1
120921..121730	hypothetical protein	120995	T	C	AE000513.1
-	-	140284	.	G	AE000513.1
-	-	140521	C	.	AE000513.1
143750..145225	hypothetical protein	145137	A	.	AE000513.1
-	-	169840	A	G	AE000513.1
188132..188953	enoyl-CoA hydratase, putative	188427	A	C	AE000513.1
194117..196531	glycogen debranching enzyme-related protein	195384	C	.	AE000513.1
194117..196531	glycogen debranching enzyme-related protein	195387	.	C	AE000513.1

complement(224979..225779)	phosphoribosylformylglycinamidine synthase I	225655	C	.	AE000513.1
231290..231487	transcriptional regulator, ArsR family	231484	T	.	AE000513.1
-	-	236338	.	G	AE000513.1
-	-	247886	.	G	AE000513.1
-	-	250349	G	A	AE000513.1
-	-	267531	.	C	AE000513.1
-	-	269001	.	C	AE000513.1
280898..281992	branched-chain amino acid ABC transporter	281968	.	G	AE000513.1
-	-	301273	.	G	AE000513.1
complement(305418..306878)	hypothetical protein	305508	.	C	AE000513.1
complement(321592..322686)	periplasmic serine protease, HtrA/DegQ/Deg	322529	.	G	AE000513.1
326390..328327	conserved hypothetical protein	327573	A	G	AE000513.1
331099..332535	ATP-dependent RNA helicase, putative	331122	C	.	AE000513.1
-	-	336213	.	G	AE000513.1
complement(336254..336640)	hypothetical protein	336433	T	A	AE000513.1

-	-	352110	.	G	AE000513.1
complement(352185..354467)	ribonuclease	352233	.	G	AE000513.1
complement(352185..354467)	ribonuclease	353191	.	G	AE000513.1
complement(352185..354467)	ribonuclease	353217	.	G	AE000513.1
complement(352185..354467)	ribonuclease	353231	.	G	AE000513.1
-	-	354488	.	C	AE000513.1
-	-	360337	.	G	AE000513.1
360595..361422	competence protein ComEC/Rec2-related protein	360664	.	G	AE000513.1
-	-	380852	.	C	AE000513.1
-	-	381907	.	C	AE000513.1
-	-	394777	C	.	AE000513.1
-	-	394898	.	C	AE000513.1
-	-	394898	.	G	AE000513.1
complement(401573..404515)	cell division protein FtsK, putative	403466	G	.	AE000513.1
complement(401573..404515)	cell division protein FtsK, putative	403475	T	.	AE000513.1
complement(401573..404515)	cell division protein FtsK, putative	403481	G	.	AE000513.1
complement(401573..404515)	cell division protein FtsK, putative	404047	G	C	AE000513.1

complement(401573..404515)	cell division protein FtsK, putative	404048	C	G	AE000513.1
complement(404533..405204)	hypothetical protein	404566	.	G	AE000513.1
-	-	415423	.	C	AE000513.1
-	-	433591	.	C	AE000513.1
-	-	433615	.	C	AE000513.1
437368..437868	conserved hypothetical protein	437802	.	G	AE000513.1
-	-	442092	.	G	AE000513.1
-	-	461682	G	T	AE000513.1
-	-	461684	A	C	AE000513.1
-	-	461685	T	C	AE000513.1
-	-	461686	Y	G	AE000513.1
-	-	461689	.	C	AE000513.1
complement(461814..463616)	maltooligosyltrehalose trehalohydrolase	462329	.	C	AE000513.1
complement(461814..463616)	maltooligosyltrehalose trehalohydrolase	462329	.	G	AE000513.1
complement(461814..463616)	maltooligosyltrehalose trehalohydrolase	462329	.	T	AE000513.1
complement(461814..463616)	maltooligosyltrehalose trehalohydrolase	462329	.	C	AE000513.1

complement(461814..463616)	maltooligosyltrehalose trehalohydrolase	462329	.	A	AE000513.1
complement(461814..463616)	maltooligosyltrehalose trehalohydrolase	462329	.	G	AE000513.1
complement(469213..469884)	hypothetical protein	469235	.	G	AE000513.1
477580..479166	sodium:solute symporter protein	477940	A	G	AE000513.1
complement(480753..483374)	penicillin-binding protein 1	481147	.	G	AE000513.1
complement(480753..483374)	penicillin-binding protein 1	481211	K	.	AE000513.1
complement(480753..483374)	penicillin-binding protein 1	481216	.	C	AE000513.1
-	-	492795	G	.	AE000513.1
complement(515856..517352)	ABC transporter, ATP-binding protein	516961	.	G	AE000513.1
complement(515856..517352)	ABC transporter, ATP-binding protein	516961	.	C	AE000513.1
complement(515856..517352)	ABC transporter, ATP-binding protein	516984	.	G	AE000513.1

complement(556197..557441)	glutaryl-CoA dehydrogenase, putative	556379	C	T	AE000513.1
581498..584413	hypothetical protein	583819	T	C	AE000513.1
-	-	593187	.	C	AE000513.1
621293..621781	hypothetical protein	621468	.	C	AE000513.1
659879..660640	hypothetical protein	660559	.	C	AE000513.1
668543..669382	serine esterase, putative	669378	G	.	AE000513.1
complement(734351..735799)	leucyl aminopeptidase, putative	735479	.	G	AE000513.1
750380..750790	hypothetical protein	750763	.	G	AE000513.1
751046..751687	hypothetical protein	751061	T	.	AE000513.1
751046..751687	hypothetical protein	751611	.	C	AE000513.1
753043..754206	conserved hypothetical protein	753158	.	G	AE000513.1
753043..754206	conserved hypothetical protein	753177	T	G	AE000513.1
753043..754206	conserved hypothetical protein	753247	C	G	AE000513.1
-	-	754376	.	G	AE000513.1
-	-	776642	.	G	AE000513.1
-	-	814241	A	.	AE000513.1

complement(815859..816257)	hypothetical protein	816173	.	G	AE000513.1
complement(816355..816597)	hypothetical protein	816405	.	G	AE000513.1
865304..867274	hypothetical protein	867147	.	C	AE000513.1
868270..869127	hypothetical protein	868397	.	C	AE000513.1
-	-	869171	.	C	AE000513.1
-	-	895026	.	G	AE000513.1
complement(910719..911828)	hypothetical protein	910800	.	G	AE000513.1
927296..927649	hypothetical protein	927619	.	G	AE000513.1
-	-	927650	.	C	AE000513.1
complement(928796..929470)	hypothetical protein	929192	.	C	AE000513.1
945063..945758	hypothetical protein	945148	.	C	AE000513.1
953899..955086	hypothetical protein	954070	.	C	AE000513.1
953899..955086	hypothetical protein	954070	.	C	AE000513.1
953899..955086	hypothetical protein	954071	.	C	AE000513.1
980855..984631	5-methyltetrahydrofolate--homocystein	983878	.	C	AE000513.1
980855..984631	5-methyltetrahy	983878	.	G	AE000513.1

	drofolate--homocystein				
980855..984631	5-methyltetrahydrofolate--homocystein	983878	.	T	AE000513.1
980855..984631	5-methyltetrahydrofolate--homocystein	983878	.	G	AE000513.1
980855..984631	5-methyltetrahydrofolate--homocystein	983878	.	A	AE000513.1
980855..984631	5-methyltetrahydrofolate--homocystein	983878	.	A	AE000513.1
996456..997634	glutamate dehydrogenase, putative	997442	.	C	AE000513.1
996456..997634	glutamate dehydrogenase, putative	997565	.	C	AE000513.1
-	-	1000824	.	C	AE000513.1
-	-	1001226	.	G	AE000513.1
1003017..1004996	extracellular solute binding protein, family 5	1003214	C	.	AE000513.1
1003017..1004996	extracellular solute binding protein, family 5	1003215	A	.	AE000513.1
1003017..1004996	extracellular solute binding	1003216	A	.	AE000513.1

	protein, family 5				
1003017..1004996	extracellular solute binding protein, family 5	1003217	G	.	AE000513.1
1003017..1004996	extracellular solute binding protein, family 5	1003218	A	.	AE000513.1
-	-	1008005	.	G	AE000513.1
1012367..1013149	transcriptional regulator, FNR/CRP family	1012909	G	.	AE000513.1
1022660..1023292	hypothetical protein	1022722	.	C	AE000513.1
1022660..1023292	hypothetical protein	1022726	.	C	AE000513.1
1022660..1023292	hypothetical protein	1022896	.	G	AE000513.1
1022660..1023292	hypothetical protein	1022898	.	G	AE000513.1
1022660..1023292	hypothetical protein	1022954	.	G	AE000513.1
complement(1026744..1027598)	hypothetical protein	1026821	.	C	AE000513.1
1037055..1037588	hypothetical protein	1037076	.	G	AE000513.1
-	-	1040283	.	G	AE000513.1
1041530..1042015	epoxidase, putative	1041878	.	G	AE000513.1
complement(1050030..1051211)	branched-chain amino	1050363	G	.	AE000513.1

	acid ABC transporter				
-	-	1052088	A	G	AE000513.1
-	-	1052234	.	C	AE000513.1
-	-	1052411	.	G	AE000513.1
1056019..1056357	hypothetical protein	1056348	.	C	AE000513.1
complement(1064215..1064925)	hydrolase, putative	1064290	.	G	AE000513.1
complement(1069192..1070088)	hypothetical protein	1069194	.	G	AE000513.1
complement(1069192..1070088)	hypothetical protein	1069708	G	.	AE000513.1
complement(1069192..1070088)	hypothetical protein	1069709	C	.	AE000513.1
complement(1069192..1070088)	hypothetical protein	1069710	G	.	AE000513.1
1071779..1072945	NADPH quinone oxidoreductase, putative	1071779	.	C	AE000513.1
1071779..1072945	NADPH quinone oxidoreductase, putative	1071829	.	C	AE000513.1
-	-	1073121	.	G	AE000513.1
complement(1075873..1076655)	hypothetical protein	1076215	G	.	AE000513.1
-	-	1077277	C	.	AE000513.1

1081696..1082061	hypothetical protein	1082021	.	C	AE000513.1
1082045..1082800	(3R)-hydroxymyristoyl-acyl carrier protei	1082259	C	.	AE000513.1
complement(1099503..1099988)	hypothetical protein	1099518	C	.	AE000513.1
-	-	1100642	.	C	AE000513.1
-	-	1100740	.	C	AE000513.1
-	-	1106348	.	G	AE000513.1
1110055..1112328	ABC transporter, ATP-binding protein, EF-	1112028	.	G	AE000513.1
-	-	1112537	C	.	AE000513.1
complement(1125852..1126874)	conserved hypothetical protein	1125976	G	A	AE000513.1
1129614..1130732	prephenate dehydrogenase	1130547	C	G	AE000513.1
-	-	1134710	.	G	AE000513.1
1134718..1136772	single-stranded-DNA-specific exonuclease	1136609	.	G	AE000513.1
-	-	1145169	.	G	AE000513.1
complement(1147461..1148225)	hypothetical protein	1147504	.	C	AE000513.1
complement(1152328..1153038)	hypothetical protein	1152406	.	C	AE000513.1

1157470..1157997	conserved hypothetical protein	1157628	.	C	AE000513.1
1157470..1157997	conserved hypothetical protein	1157628	.	T	AE000513.1
1157470..1157997	conserved hypothetical protein	1157628	.	G	AE000513.1
-	-	1159254	G	.	AE000513.1
complement(1161195..1162103)	3-hydroxybutyryl-CoA dehydratase	1161635	A	G	AE000513.1
-	-	1168428	.	G	AE000513.1
-	-	1185927	.	C	AE000513.1
1205621..1205929	hypothetical protein	1205870	T	.	AE000513.1
1205621..1205929	hypothetical protein	1205871	C	G	AE000513.1
1205621..1205929	hypothetical protein	1205872	C	G	AE000513.1
1205621..1205929	hypothetical protein	1205873	C	A	AE000513.1
1205621..1205929	hypothetical protein	1205874	C	T	AE000513.1
1209784..1212849	hypothetical protein	1211737	A	G	AE000513.1
complement(1216195..1217997)	cell cycle protein MesJ, putative/cytosin	1217013	C	.	AE000513.1
1219673..1220260	hypothetical protein	1220133	T	.	AE000513.1
1232894..1233985	hypothetical protein	1233932	.	C	AE000513.1

1235582..1235986	hypothetical protein	1235591	G	.	AE000513.1
complement(1235983..1236942)	hypothetical protein	1236732	.	G	AE000513.1
1265444..1267246	DNA helicase, SNF2/RAD54 family	1265521	C	.	AE000513.1
1275795..1276199	hypothetical protein	1275805	.	C	AE000513.1
1275795..1276199	hypothetical protein	1275869	.	G	AE000513.1
-	-	1277438	.	G	AE000513.1
1291027..1291446	hypothetical protein	1291068	.	C	AE000513.1
complement(1292534..1293145)	conserved hypothetical protein	1292598	.	C	AE000513.1
complement(1307372..1308067)	hypothetical protein	1307999	.	G	AE000513.1
complement(1307372..1308067)	hypothetical protein	1308020	.	G	AE000513.1
complement(1307372..1308067)	hypothetical protein	1308021	T	C	AE000513.1
complement(1307372..1308067)	hypothetical protein	1308026	S	A	AE000513.1
complement(1307372..1308067)	hypothetical protein	1308028	.	G	AE000513.1
complement(1307372..1308067)	hypothetical protein	1308029	A	C	AE000513.1

complement(1307372..1308067)	hypothetical protein	1308030	G	A	AE000513.1
complement(1307372..1308067)	hypothetical protein	1308034	A	.	AE000513.1
-	-	1318403	.	G	AE000513.1
1323043..1323279	hypothetical protein	1323074	T	C	AE000513.1
1323043..1323279	hypothetical protein	1323075	G	T	AE000513.1
1323454..1324650	acyl-CoA dehydrogenase	1324318	C	G	AE000513.1
1323454..1324650	acyl-CoA dehydrogenase	1324319	G	C	AE000513.1
-	-	1327764	.	C	AE000513.1
-	-	1327828	G	.	AE000513.1
1332428..1333861	multidrug-efflux transporter	1333766	.	G	AE000513.1
complement(1334225..1335019)	conserved hypothetical protein	1334807	A	.	AE000513.1
complement(1334225..1335019)	conserved hypothetical protein	1334811	.	C	AE000513.1
complement(1336292..1336690)	hypothetical protein	1336356	.	G	AE000513.1
complement(1336292..1336690)	hypothetical protein	1336376	C	.	AE000513.1

complement(1336760..1337806)	glycosyl hyrolase, family 3	1336760	T	C	AE000513.1
1342520..1344181	transport protein, putative	1342753	.	G	AE000513.1
1342520..1344181	transport protein, putative	1343009	.	G	AE000513.1
1350424..1351092	hydrolase	1350441	C	.	AE000513.1
1351086..1351622	hypothetical protein	1351524	.	C	AE000513.1
1363325..1364302	ABC transporter, ATP-binding protein	1363632	C	G	AE000513.1
1378605..1381670	DNA topoisomerase I	1378670	.	C	AE000513.1
complement(1406927..1407250)	hypothetical protein	1406938	.	G	AE000513.1
complement(1408946..1409383)	hypothetical protein	1409347	G	.	AE000513.1
-	-	1417882	.	G	AE000513.1
-	-	1417884	G	C	AE000513.1
complement(1418582..1421611)	penicillin-binding protein 1B McrB, putative	1419293	.	G	AE000513.1
-	-	1423853	A	G	AE000513.1
-	-	1443139	.	G	AE000513.1

complement(1451400..1452680)	hypothetical protein	1451624	.	C	AE000513.1
-	-	1456205	C	T	AE000513.1
-	-	1456209	C	T	AE000513.1
-	-	1456212	A	G	AE000513.1
-	-	1456216	A	G	AE000513.1
-	-	1456219	T	C	AE000513.1
-	-	1460634	A	.	AE000513.1
complement(1465188..1471010)	hypothetical protein	1465338	.	G	AE000513.1
complement(1465188..1471010)	hypothetical protein	1468015	G	C	AE000513.1
complement(1465188..1471010)	hypothetical protein	1470524	.	G	AE000513.1
complement(1470856..1475187)	hypothetical protein	1470933	.	C	AE000513.1
-	-	1475208	.	G	AE000513.1
-	-	1475209	.	G	AE000513.1
-	-	1481060	.	C	AE000513.1
-	-	1481088	.	C	AE000513.1
complement(1498936..1499907)	lipase, putative	1499059	.	C	AE000513.1
complement(1498936..1499907)	lipase, putative	1499091	.	G	AE000513.1
complement(1498936..1499907)	lipase, putative	1499091	.	C	AE000513.1
-	-	1504300	.	G	AE000513.1

complement(1509948..1511885)	NADH dehydrogenase I, L subunit	1510739	C	.	AE000513.1
-	-	1533823	.	C	AE000513.1
-	-	1538253	.	G	AE000513.1
1543319..1543720	hypothetical protein	1543632	.	G	AE000513.1
complement(1566493..1568223)	ftsE protein	1567364	.	G	AE000513.1
complement(1566493..1568223)	ftsE protein	1567364	.	C	AE000513.1
complement(1566493..1568223)	ftsE protein	1567366	G	C	AE000513.1
-	-	1572134	.	G	AE000513.1
-	-	1572569	.	C	AE000513.1
1577968..1578534	hypothetical protein	1578013	.	G	AE000513.1
complement(1587670..1588800)	peptide ABC transporter, permease protein	1587965	A	C	AE000513.1
-	-	1593381	.	G	AE000513.1
complement(1605887..1606996)	glutamate racemase	1606957	.	C	AE000513.1
1621524..1622936	conserved hypothetical protein	1622844	.	C	AE000513.1
-	-	1628748	.	C	AE000513.1
-	-	1644529	C	.	AE000513.1
-	-	1644543	T	.	AE000513.1
-	-	1645961	.	G	AE000513.1

-	-	1651396	.	G	AE000513.1
-	-	1656768	A	G	AE000513.1
-	-	1656769	G	A	AE000513.1
complement(1660103..1661395)	hypothetical protein	1661111	.	G	AE000513.1
complement(1660103..1661395)	hypothetical protein	1661204	.	G	AE000513.1
complement(1660103..1661395)	hypothetical protein	1661207	.	C	AE000513.1
-	-	1661416	.	C	AE000513.1
-	-	1661493	.	G	AE000513.1
-	-	1664674	.	G	AE000513.1
1666372..1666692	hypothetical protein	1666629	.	C	AE000513.1
1666372..1666692	hypothetical protein	1666629	.	A	AE000513.1
1666372..1666692	hypothetical protein	1666629	.	G	AE000513.1
1668196..1668828	hypothetical protein	1668212	.	C	AE000513.1
-	-	1671409	.	C	AE000513.1
1680022..1681581	Mg(2+) chelatase family protein	1681486	T	G	AE000513.1
complement(1771768..1772997)	fosmidomycin resistance protein, putative	1772409	C	T	AE000513.1
-	-	1776081	.	C	AE000513.1
1814915..1816354	mocR protein	1815401	.	G	AE000513.1

1814915..1816354	mocR protein	1815426	G	.	AE000513.1
1816987..1818003	ribosomal large subunit pseudouridine synthas	1817033	C	.	AE000513.1
-	-	1829905	.	G	AE000513.1
-	-	1829908	G	C	AE000513.1
-	-	1838361	.	G	AE000513.1
-	-	1840322	.	G	AE000513.1
complement(1883538..1883777)	hypothetical protein	1883541	C	.	AE000513.1
-	-	1888789	C	.	AE000513.1
1892037..1893227	chloromuconate cycloisomerase, putative	1892723	C	G	AE000513.1
1892037..1893227	chloromuconate cycloisomerase, putative	1892724	G	A	AE000513.1
1892037..1893227	chloromuconate cycloisomerase, putative	1892727	G	C	AE000513.1
complement(1904512..1905669)	transcriptional regulator	1904580	C	.	AE000513.1
1906346..1906921	conserved hypothetical protein	1906884	.	G	AE000513.1
-	-	1928137	.	C	AE000513.1
-	-	1935000	.	G	AE000513.1
-	-	1935234	.	C	AE000513.1

1935271..1935453	hypothetical protein	1935418	.	G	AE000513.1
1942746..1945475	exonuclease SbcC	1943461	.	C	AE000513.1
1942746..1945475	exonuclease SbcC	1943472	.	G	AE000513.1
1942746..1945475	exonuclease SbcC	1943474	K	A	AE000513.1
1942746..1945475	exonuclease SbcC	1943484	T	G	AE000513.1
1942746..1945475	exonuclease SbcC	1943485	.	G	AE000513.1
1942746..1945475	exonuclease SbcC	1943531	T	C	AE000513.1
1942746..1945475	exonuclease SbcC	1944586	.	G	AE000513.1
1942746..1945475	exonuclease SbcC	1944594	.	G	AE000513.1
1942746..1945475	exonuclease SbcC	1944594	.	C	AE000513.1
1942746..1945475	exonuclease SbcC	1944809	A	G	AE000513.1
1942746..1945475	exonuclease SbcC	1944810	G	C	AE000513.1
1945630..1946460	hypothetical protein	1946367	.	C	AE000513.1
-	-	1946473	.	C	AE000513.1
complement(1952451..1953236)	conserved hypothetical protein	1952485	.	C	AE000513.1
complement(1963590..1964876)	3-oxoacyl-acyl carrier protein synthase II	1963745	G	C	AE000513.1
complement(1963590..1964876)	3-oxoacyl-acyl carrier protein synthase II	1963746	C	G	AE000513.1

1981234..1981764	ankyrin-related protein	1981601	.	G	AE000513.1
1994173..1996614	ATP-dependent protease LA	1995430	G	T	AE000513.1
1994173..1996614	ATP-dependent protease LA	1995441	C	G	AE000513.1
complement(2007783..2008421)	thymidine kinase	2007847	.	C	AE000513.1
complement(2007783..2008421)	thymidine kinase	2007847	.	G	AE000513.1
complement(2013610..2014671)	conserved hypothetical protein	2014654	.	C	AE000513.1
complement(2013610..2014671)	conserved hypothetical protein	2014654	.	G	AE000513.1
-	-	2016380	C	.	AE000513.1
-	-	2018608	A	.	AE000513.1
-	-	2022950	.	G	AE000513.1
-	-	2031960	G	A	AE000513.1
-	-	2031964	G	C	AE000513.1
-	-	2031967	A	.	AE000513.1
-	-	2031968	G	C	AE000513.1
-	-	2031969	G	T	AE000513.1
-	-	2046695	.	G	AE000513.1
complement(2047650..2050013)	glutamine synthase	2048322	G	.	AE000513.1
complement(2047650..2050013)	glutamine synthase	2048325	.	G	AE000513.1

complement(2056384..2058351)	conserved hypothetical protein	2056841	.	G	AE000513.1
complement(2056384..2058351)	conserved hypothetical protein	2056841	.	C	AE000513.1
complement(2062196..2063413)	elongation factor TU	2062775	G	.	AE000513.1
-	-	2068922	.	G	AE000513.1
-	-	2068940	.	G	AE000513.1
-	-	2069318	.	G	AE000513.1
-	-	2069323	.	C	AE000513.1
2072204..2073190	hypothetical protein	2073092	.	C	AE000513.1
2101779..2103440	hypothetical protein	2101879	T	C	AE000513.1
complement(2135694..2136146)	hypothetical protein	2135920	T	G	AE000513.1
complement(2136586..2138172)	conserved hypothetical protein	2137189	.	C	AE000513.1
complement(2144834..2145868)	conserved hypothetical protein	2144907	T	.	AE000513.1
complement(2144834..2145868)	conserved hypothetical protein	2144910	C	G	AE000513.1
2146441..2148099	ABC transporter, ATP-binding protein	2147407	.	C	AE000513.1
-	-	2148109	.	C	AE000513.1
-	-	2148895	.	G	AE000513.1

complement(2149081..2149761)	hypothetical protein	2149109	C	G	AE000513.1
complement(2149081..2149761)	hypothetical protein	2149110	.	C	AE000513.1
-	-	2150439	G	.	AE000513.1
-	-	2157569	.	G	AE000513.1
-	-	2157569	.	G	AE000513.1
2160561..2160971	hypothetical protein	2160661	G	C	AE000513.1
-	-	2188925	C	.	AE000513.1
2189795..2190067	hypothetical protein	2189942	G	A	AE000513.1
complement(2194404..2195789)	hypothetical protein	2194956	N	.	AE000513.1
complement(2194404..2195789)	hypothetical protein	2194958	.	G	AE000513.1
2202100..2203284	UDP-glucose 4-epimerase	2203078	.	G	AE000513.1
-	-	2215178	.	C	AE000513.1
complement(2231724..2232062)	hypothetical protein	2231823	.	G	AE000513.1
-	-	2238344	.	C	AE000513.1
-	-	2238474	.	C	AE000513.1
2242761..2244233	methoxyneurosporene dehydrogenase	2244231	T	C	AE000513.1
-	-	2247372	G	.	AE000513.1
-	-	2314060	C	G	AE000513.1

complement(2339068..2340492)	birA bifunctional protein	2340149	.	G	AE000513.1
2346058..2347737	hypothetical protein	2347390	T	C	AE000513.1
2347866..2348678	hypothetical protein	2348609	.	C	AE000513.1
-	-	2365230	.	G	AE000513.1
complement(2365388..2367115)	glutathione-regulated potassium-efflux syste	2365390	.	G	AE000513.1
complement(2365388..2367115)	glutathione-regulated potassium-efflux syste	2365409	A	C	AE000513.1
complement(2401695..2402672)	conserved hypothetical protein	2402411	.	G	AE000513.1
2410637..2412484	DNA polymerase III, tau/gamma subunit	2412461	T	.	AE000513.1
-	-	2420014	A	.	AE000513.1
2421284..2422405	DNA-binding response regulator	2421928	.	G	AE000513.1
2421284..2422405	DNA-binding response regulator	2422010	.	C	AE000513.1
-	-	2431599	C	.	AE000513.1
2433075..2434337	conserved hypothetical protein	2434200	C	.	AE000513.1
-	-	2435646	.	C	AE000513.1
-	-	2437558	C	.	AE000513.1

2445791..2446162	hypothetical protein	2446149	.	G	AE000513.1
2469804..2470949	cytochrome P450	2470471	T	C	AE000513.1
complement(2471534..2472199)	hypothetical protein	2471633	A	.	AE000513.1
-	-	2474213	.	G	AE000513.1
-	-	2474542	.	G	AE000513.1
2484833..2486380	ABC transporter, permease protein, CysTW family	2486190	T	C	AE000513.1
2486796..2488229	hypothetical protein	2486934	.	G	AE000513.1
2486796..2488229	hypothetical protein	2488045	T	C	AE000513.1
-	-	2490732	.	C	AE000513.1
complement(2508643..2511489)	hexagonally packed intermediate-layer surfac	2511220	G	A	AE000513.1
2512346..2513128	enoyl-CoA hydratase, putative	2513107	G	A	AE000513.1
-	-	2517684	.	C	AE000513.1
complement(2517685..2518812)	hypothetical protein	2518676	.	G	AE000513.1
-	-	2525930	.	C	AE000513.1
complement(2532961..2533398)	hypothetical protein	2533038	C	.	AE000513.1
-	-	2533406	.	G	AE000513.1

2533937..2534788	thiosulfate sulfurtransferase	2534551	T	C	AE000513.1
2537848..2538933	conserved hypothetical protein	2538821	.	C	AE000513.1
complement(2547403..2549442)	conserved hypothetical protein	2548730	T	C	AE000513.1
2560973..2561443	hypothetical protein	2561376	.	G	AE000513.1
2567723..2568229	hypothetical protein	2567892	.	C	AE000513.1
-	-	2569064	G	C	AE000513.1
complement(2572383..2573195)	hypothetical protein	2573194	.	G	AE000513.1
-	-	2573225	.	G	AE000513.1
-	-	2573340	.	G	AE000513.1
2574755..2576245	hypothetical protein	2576197	.	C	AE000513.1
2576233..2576544	hypothetical protein	2576358	A	C	AE000513.1
-	-	2577432	G	C	AE000513.1
-	-	2577471	.	G	AE000513.1
-	-	2577471	.	C	AE000513.1
-	-	2577492	.	G	AE000513.1
complement(2590036..2590593)	conserved hypothetical protein	2590080	.	C	AE000513.1
-	-	2590637	.	C	AE000513.1
2594387..2595028	conserved hypothetical protein	2594406	A	G	AE000513.1
-	-	2615343	A	C	AE000513.1

complement(2616672..2617097)	hypothetical protein	2616866	G	T	AE000513.1
-	-	2628828	.	C	AE000513.1
-	-	19081	.	A	AE001825.1
-	-	26635	.	G	AE001825.1
-	-	27198	.	G	AE001825.1
complement(56537..56779)	hypothetical protein	56564	.	G	AE001825.1
complement(79518..80240)	hypothetical protein	79628	.	G	AE001825.1
91974..92366	hypothetical protein	91974	.	C	AE001825.1
91974..92366	hypothetical protein	92323	G	.	AE001825.1
-	-	95204	.	C	AE001825.1
97981..98715	hypothetical protein	98329	.	G	AE001825.1
97981..98715	hypothetical protein	98329	.	C	AE001825.1
102993..105422	hypothetical protein	103187	.	C	AE001825.1
complement(126441..127172)	hypothetical protein	126646	.	C	AE001825.1
complement(132390..133382)	acyl-CoA dehydrogenase, putative	132435	.	C	AE001825.1
complement(133289..134692)	hypothetical protein	133390	.	C	AE001825.1
161935..162603	phosphate transport system regulatory protei	162113	A	G	AE001825.1

184027..184851	conserved hypothetical protein	184065	.	G	AE001825.1
-	-	193733	.	G	AE001825.1
197036..198550	conserved hypothetical protein	197080	C	.	AE001825.1
-	-	220602	.	C	AE001825.1
-	-	222815	A	.	AE001825.1
-	-	225269	A	.	AE001825.1
227890..228774	conserved hypothetical protein	228597	.	G	AE001825.1
-	-	281718	.	C	AE001825.1
-	-	302667	G	C	AE001825.1
-	-	302894	T	C	AE001825.1
-	-	303032	.	C	AE001825.1
303497..303838	hypothetical protein	303753	.	G	AE001825.1
303829..305343	hypothetical protein	305178	C	G	AE001825.1
308594..309277	conserved hypothetical protein	309263	.	C	AE001825.1
308594..309277	conserved hypothetical protein	309263	.	G	AE001825.1
-	-	309515	.	C	AE001825.1
complement(311654..312718)	hypothetical protein	312711	.	G	AE001825.1
316139..316645	hypothetical protein	316621	.	G	AE001825.1
316696..319737	hypothetical protein	319681	.	C	AE001825.1

complement(320651..320992)	hypothetical protein	320768	.	C	AE001825.1
-	-	321029	.	G	AE001825.1
complement(321078..321617)	hypothetical protein	321170	.	G	AE001825.1
complement(323604..324353)	hypothetical protein	323973	G	C	AE001825.1
complement(329200..331329)	hypothetical protein	329439	.	G	AE001825.1
complement(329200..331329)	hypothetical protein	329439	.	C	AE001825.1
complement(331332..332087)	hypothetical protein	331334	.	G	AE001825.1
complement(340933..341400)	urease accessory protein UreE	341356	A	G	AE001825.1
356999..358186	hypothetical protein	358104	.	C	AE001825.1
390277..392547	methyl-accepting chemotaxis-related protein	392473	.	G	AE001825.1
390277..392547	methyl-accepting chemotaxis-related protein	392476	G	.	AE001825.1
-	-	395959	.	C	AE001825.1
-	-	26999	.	G	AE001826.1
27010..28065	TorS-related protein	27215	G	.	AE001826.1

41751..42122	hypothetical protein	42095	.	G	AE001826.1
44540..44977	hypothetical protein	44935	G	.	AE001826.1
44988..47798	hypothetical protein	46222	T	C	AE001826.1
44988..47798	hypothetical protein	46275	T	C	AE001826.1
44988..47798	hypothetical protein	46279	.	G	AE001826.1
44988..47798	hypothetical protein	46301	G	C	AE001826.1
44988..47798	hypothetical protein	46303	C	G	AE001826.1
44988..47798	hypothetical protein	46304	.	C	AE001826.1
44988..47798	hypothetical protein	46326	.	G	AE001826.1
-	-	94328	.	C	AE001826.1
complement(99585..100214)	DNA-binding response regulator	99823	T	C	AE001826.1
-	-	106291	C	.	AE001826.1
complement(176244..177437)	hypothetical protein	176400	.	G	AE001826.1
73..384	cytochrome P450-related protein	145	.	C	AE001827.1
-	-	594	.	C	AE001827.1
-	-	596	T	C	AE001827.1
-	-	3119	.	G	AE001827.1
-	-	3595	C	T	AE001827.1
-	-	8096	.	G	AE001827.1

complement(8308..10218)	hypothetical protein	9122	G	C	AE001827.1
-	-	12161	.	G	AE001827.1
-	-	12480	.	C	AE001827.1
-	-	12483	.	C	AE001827.1
-	-	12499	C	T	AE001827.1
13519..14157	N-acetylmuramoyl-L-alanine amidase, putative	14100	.	C	AE001827.1
-	-	14158	A	.	AE001827.1
-	-	14189	T	.	AE001827.1
-	-	14190	N	.	AE001827.1
complement(16040..16852)	hypothetical protein	16189	.	C	AE001827.1
-	-	17763	.	C	AE001827.1
-	-	22069	.	C	AE001827.1
complement(23296..23892)	hypothetical protein	23302	.	G	AE001827.1
-	-	24164	.	C	AE001827.1
-	-	24401	C	.	AE001827.1
-	-	24874	.	A	AE001827.1
-	-	24903	.	C	AE001827.1
24950..25609	hypothetical protein	25499	T	.	AE001827.1
-	-	25711	.	C	AE001827.1
27711..28106	hypothetical protein	27911	.	C	AE001827.1
30272..31138	transposase, putative	30415	G	T	AE001827.1
30272..31138	transposase, putative	30418	C	G	AE001827.1

30272..31138	transposase, putative	30442	T	C	AE001827.1
30272..31138	transposase, putative	30451	T	C	AE001827.1
30272..31138	transposase, putative	30457	C	G	AE001827.1
30272..31138	transposase, putative	30463	T	C	AE001827.1
30272..31138	transposase, putative	30466	A	G	AE001827.1
30272..31138	transposase, putative	30487	C	G	AE001827.1
30272..31138	transposase, putative	30535	G	T	AE001827.1
30272..31138	transposase, putative	30541	C	T	AE001827.1
30272..31138	transposase, putative	30547	C	T	AE001827.1
30272..31138	transposase, putative	30550	G	A	AE001827.1
30272..31138	transposase, putative	30568	T	C	AE001827.1
30272..31138	transposase, putative	30587	A	C	AE001827.1
30272..31138	transposase, putative	30592	A	G	AE001827.1
30272..31138	transposase, putative	30595	T	C	AE001827.1
30272..31138	transposase, putative	30598	T	C	AE001827.1
30272..31138	transposase, putative	30619	A	G	AE001827.1
30272..31138	transposase, putative	30631	G	T	AE001827.1

30272..31138	transposase, putative	30673	C	T	AE001827.1
30272..31138	transposase, putative	30679	T	C	AE001827.1
30272..31138	transposase, putative	30681	G	A	AE001827.1
30272..31138	transposase, putative	30724	A	G	AE001827.1
30272..31138	transposase, putative	30749	T	G	AE001827.1
30272..31138	transposase, putative	30750	C	T	AE001827.1
30272..31138	transposase, putative	30789	G	A	AE001827.1
30272..31138	transposase, putative	30799	A	G	AE001827.1
30272..31138	transposase, putative	30814	T	A	AE001827.1
30272..31138	transposase, putative	30832	T	C	AE001827.1
30272..31138	transposase, putative	30838	A	G	AE001827.1
30272..31138	transposase, putative	30850	T	C	AE001827.1
30272..31138	transposase, putative	30854	T	C	AE001827.1
30272..31138	transposase, putative	30856	A	G	AE001827.1
30272..31138	transposase, putative	30862	A	G	AE001827.1
30272..31138	transposase, putative	30865	C	T	AE001827.1
30272..31138	transposase, putative	30904	A	C	AE001827.1

30272..31138	transposase, putative	30913	A	G	AE001827.1
30272..31138	transposase, putative	30925	G	A	AE001827.1
30272..31138	transposase, putative	30932	T	C	AE001827.1
30272..31138	transposase, putative	30937	C	A	AE001827.1
30272..31138	transposase, putative	30940	G	T	AE001827.1
30272..31138	transposase, putative	30952	G	C	AE001827.1
30272..31138	transposase, putative	30962	T	C	AE001827.1
30272..31138	transposase, putative	30988	T	C	AE001827.1
30272..31138	transposase, putative	30991	G	C	AE001827.1
30272..31138	transposase, putative	31010	C	A	AE001827.1
30272..31138	transposase, putative	31075	A	G	AE001827.1
30272..31138	transposase, putative	31087	A	T	AE001827.1
30272..31138	transposase, putative	31091	C	G	AE001827.1
-	-	33781	.	C	AE001827.1
-	-	34155	.	C	AE001827.1
-	-	35076	.	G	AE001827.1
-	-	42608	.	C	AE001827.1

Supplementary Table S2. Annotations of genes affected by SNPs and indels between two *D. radiodurans* strains – genome-1999 (see Methods for details) was used as the reference DNA sequence for this comparison, and genome-2016 as the query.

Coordinates of the gene with the SNP/inde	Function of the gene with the SNP/indel	Position of the snp in the reference	Character in the reference	Character in the query	Reference fasta id
complement(1..1182)	DNA polymerase III, beta subunit	145	G	.	AE000513.1
-	-	1618	A	.	AE000513.1
complement(18182..19063)	hypothetical protein	18902	G	.	AE000513.1
19258..20643	ribonuclease II family protein	20505	G	.	AE000513.1
37446..38756	serine hydroxymethyltransferase	37941	A	G	AE000513.1
41050..41823	hypothetical protein	41475	C	.	AE000513.1
-	-	54429	C	.	AE000513.1
55152..55580	hypothetical protein	55517	G	.	AE000513.1
-	-	65577	C	G	AE000513.1
-	-	65578	G	C	AE000513.1
complement(66759..68792)	peptidyl-prolyl cis-trans isomerse, putative	66949	.	C	AE000513.1
77159..78364	molybdopterin	77790	A	.	AE000513.1

	biosynthesis MoeA				
-	-	84580	G	.	AE000513.1
-	-	87869	C	.	AE000513.1
-	-	94720	G	.	AE000513.1
-	-	94727	G	.	AE000513.1
100371..100802	single-stranded DNA-binding protein	100733	.	G	AE000513.1
-	-	100960	.	G	AE000513.1
114105..114995	hypothetical protein	114697	.	G	AE000513.1
complement(116822..117385)	acetyl-CoA carboxylase, bitoin carboxyl carrie	117214	A	G	AE000513.1
complement(116822..117385)	acetyl-CoA carboxylase, bitoin carboxyl carrie	117250	T	.	AE000513.1
complement(116822..117385)	acetyl-CoA carboxylase, bitoin carboxyl carrie	117282	.	C	AE000513.1
120921..121730	hypothetical protein	120995	T	C	AE000513.1
-	-	140284	.	G	AE000513.1
-	-	140521	C	.	AE000513.1
143750..145225	hypothetical protein	145137	A	.	AE000513.1
-	-	169840	A	G	AE000513.1

188132..188953	enoyl-CoA hydratase, putative	188427	A	C	AE000513.1
complement(192257..192814)	thiol:disulfide interchange protein	192733	C	.	AE000513.1
194117..196531	glycogen debranching enzyme-related protein	195384	C	.	AE000513.1
194117..196531	glycogen debranching enzyme-related protein	195387	.	C	AE000513.1
complement(224979..225779)	phosphoribosylformylglycinamidine synthase I	225655	C	.	AE000513.1
231290..231487	transcriptional regulator, ArsR family	231484	T	.	AE000513.1
-	-	236338	.	G	AE000513.1
-	-	247886	.	G	AE000513.1
-	-	250349	G	A	AE000513.1
-	-	267531	.	C	AE000513.1
-	-	269001	.	C	AE000513.1
276011..276340	conserved hypothetical protein	276243	G	.	AE000513.1
complement(280195..280863)	conserved hypothetical protein	280307	C	.	AE000513.1
280898..281992	branched-chain amino	281968	.	G	AE000513.1

	acid ABC transporter				
292117..293337	conserved hypothetical protein	292570	G	.	AE000513.1
-	-	301273	.	G	AE000513.1
complement(305418..306878)	hypothetical protein	305508	.	C	AE000513.1
306969..307364	ribosomal protein S12	307221	G	.	AE000513.1
complement(321592..322686)	periplasmic serine protease, HtrA/DegQ/Deg	322529	.	G	AE000513.1
326390..328327	conserved hypothetical protein	327573	A	G	AE000513.1
331099..332535	ATP-dependent RNA helicase, putative	331122	C	.	AE000513.1
-	-	336213	.	G	AE000513.1
complement(336254..336640)	hypothetical protein	336433	T	A	AE000513.1
-	-	352110	.	G	AE000513.1
complement(352185..354467)	ribonuclease	352233	.	G	AE000513.1
complement(352185..354467)	ribonuclease	353191	.	G	AE000513.1
complement(352185..354467)	ribonuclease	353217	.	G	AE000513.1

complement(352185..354467)	ribonuclease	353231	.	G	AE000513.1
-	-	354488	.	C	AE000513.1
-	-	360337	.	G	AE000513.1
360595..361422	competence protein ComEC/Rec2-related protein	360664	.	G	AE000513.1
371543..373120	lysyl-tRNA synthetase	371566	C	.	AE000513.1
-	-	380852	.	C	AE000513.1
-	-	381907	.	C	AE000513.1
-	-	394777	C	.	AE000513.1
-	-	394898	.	C	AE000513.1
-	-	394898	.	G	AE000513.1
complement(401573..404515)	cell division protein FtsK, putative	403466	G	.	AE000513.1
complement(401573..404515)	cell division protein FtsK, putative	403475	T	.	AE000513.1
complement(401573..404515)	cell division protein FtsK, putative	403481	G	.	AE000513.1
complement(401573..404515)	cell division protein FtsK, putative	404047	G	C	AE000513.1
complement(401573..404515)	cell division protein FtsK, putative	404048	C	G	AE000513.1
complement(404533..405204)	hypothetical protein	404566	.	G	AE000513.1
-	-	415423	.	C	AE000513.1

complement(429873..430592)	conserved hypothetical protein	430585	A	C	AE000513.1
-	-	433591	.	C	AE000513.1
-	-	433615	.	C	AE000513.1
437368..437868	conserved hypothetical protein	437802	.	G	AE000513.1
-	-	442092	.	G	AE000513.1
-	-	461682	G	T	AE000513.1
-	-	461684	A	C	AE000513.1
-	-	461685	T	C	AE000513.1
-	-	461686	Y	G	AE000513.1
-	-	461689	.	C	AE000513.1
complement(461814..463616)	maltooligosyltrehalose trehalohydrolase	462329	.	C	AE000513.1
complement(461814..463616)	maltooligosyltrehalose trehalohydrolase	462329	.	G	AE000513.1
complement(461814..463616)	maltooligosyltrehalose trehalohydrolase	462329	.	T	AE000513.1
complement(461814..463616)	maltooligosyltrehalose trehalohydrolase	462329	.	C	AE000513.1
complement(461814..463616)	maltooligosyltrehalose trehalohydrolase	462329	.	A	AE000513.1

complement(461814..463616)	maltooligosyltrehalose trehalohydrolase	462329	.	G	AE000513.1
complement(461814..463616)	maltooligosyltrehalose trehalohydrolase	463581	G	.	AE000513.1
complement(469213..469884)	hypothetical protein	469235	.	G	AE000513.1
complement(480753..483374)	penicillin-binding protein 1	481147	.	G	AE000513.1
complement(480753..483374)	penicillin-binding protein 1	481211	K	.	AE000513.1
complement(480753..483374)	penicillin-binding protein 1	481216	.	C	AE000513.1
complement(480753..483374)	penicillin-binding protein 1	481824	G	.	AE000513.1
-	-	492795	G	.	AE000513.1
503614..504843	alanyl-tRNA synthetase-related protein	503906	C	.	AE000513.1
complement(515856..517352)	ABC transporter, ATP-binding protein	516961	.	G	AE000513.1
complement(515856..517352)	ABC transporter, ATP-binding protein	516961	.	C	AE000513.1

complement(515856..517352)	ABC transporter, ATP-binding protein	516984	.	G	AE000513.1
-	-	522431	G	.	AE000513.1
complement(533860..534480)	hypothetical protein	534141	C	.	AE000513.1
-	-	549335	C	.	AE000513.1
581498..584413	hypothetical protein	583819	T	C	AE000513.1
-	-	593187	.	C	AE000513.1
621293..621781	hypothetical protein	621468	.	C	AE000513.1
638737..639333	conserved hypothetical protein	639053	C	.	AE000513.1
659879..660640	hypothetical protein	660559	.	C	AE000513.1
668543..669382	serine esterase, putative	669378	G	.	AE000513.1
complement(719805..720731)	conserved hypothetical protein	719892	T	G	AE000513.1
complement(734351..735799)	leucyl aminopeptidase, putative	735479	.	G	AE000513.1
750380..750790	hypothetical protein	750763	.	G	AE000513.1
751046..751687	hypothetical protein	751061	T	.	AE000513.1
751046..751687	hypothetical protein	751611	.	C	AE000513.1

753043..754206	conserved hypothetical protein	753158	.	G	AE000513.1
753043..754206	conserved hypothetical protein	753177	T	G	AE000513.1
753043..754206	conserved hypothetical protein	753247	C	G	AE000513.1
-	-	754376	.	G	AE000513.1
-	-	776642	.	G	AE000513.1
-	-	814241	A	.	AE000513.1
complement(815859..816257)	hypothetical protein	816173	.	G	AE000513.1
complement(816355..816597)	hypothetical protein	816405	.	G	AE000513.1
-	-	818875	C	.	AE000513.1
865304..867274	hypothetical protein	867147	.	C	AE000513.1
868270..869127	hypothetical protein	868397	.	C	AE000513.1
-	-	869171	.	C	AE000513.1
-	-	895026	.	G	AE000513.1
complement(910719..911828)	hypothetical protein	910800	.	G	AE000513.1
927296..927649	hypothetical protein	927619	.	G	AE000513.1
-	-	927650	.	C	AE000513.1
complement(928796..929470)	hypothetical protein	929192	.	C	AE000513.1

945063..945758	hypothetical protein	945148	.	C	AE000513.1
953899..955086	hypothetical protein	954070	.	C	AE000513.1
953899..955086	hypothetical protein	954070	.	C	AE000513.1
953899..955086	hypothetical protein	954071	.	C	AE000513.1
953899..955086	hypothetical protein	954224	C	.	AE000513.1
975941..977191	N-acetyl-gamma-glutamyl-phosphate reductase	976846	C	.	AE000513.1
980855..984631	5-methyltetrahydrofolate--homocystein	983878	.	C	AE000513.1
980855..984631	5-methyltetrahydrofolate--homocystein	983878	.	G	AE000513.1
980855..984631	5-methyltetrahydrofolate--homocystein	983878	.	T	AE000513.1
980855..984631	5-methyltetrahydrofolate--homocystein	983878	.	G	AE000513.1
980855..984631	5-methyltetrahydrofolate--homocystein	983878	.	A	AE000513.1
980855..984631	5-methyltetrahy	983878	.	A	AE000513.1

	drofolate--homocystein				
996456..997634	glutamate dehydrogenase, putative	997442	.	C	AE000513.1
996456..997634	glutamate dehydrogenase, putative	997565	.	C	AE000513.1
-	-	1000824	.	C	AE000513.1
-	-	1001226	.	G	AE000513.1
1003017..1004996	extracellular solute binding protein, family 5	1003214	C	.	AE000513.1
1003017..1004996	extracellular solute binding protein, family 5	1003215	A	.	AE000513.1
1003017..1004996	extracellular solute binding protein, family 5	1003216	A	.	AE000513.1
1003017..1004996	extracellular solute binding protein, family 5	1003217	G	.	AE000513.1
1003017..1004996	extracellular solute binding protein, family 5	1003218	A	.	AE000513.1
-	-	1008005	.	G	AE000513.1
1012367..1013149	transcriptional regulator, FNR/CRP family	1012909	G	.	AE000513.1
1022660..1023292	hypothetical protein	1022722	.	C	AE000513.1

1022660..1023292	hypothetical protein	1022726	.	C	AE000513.1
1022660..1023292	hypothetical protein	1022896	.	G	AE000513.1
1022660..1023292	hypothetical protein	1022898	.	G	AE000513.1
1022660..1023292	hypothetical protein	1022954	.	G	AE000513.1
1024354..1025277	ABC transporter, ATP-binding protein	1024593	C	.	AE000513.1
complement(1026744..1027598)	hypothetical protein	1026821	.	C	AE000513.1
1037055..1037588	hypothetical protein	1037076	.	G	AE000513.1
-	-	1040283	.	G	AE000513.1
1041530..1042015	epoxidase, putative	1041878	.	G	AE000513.1
-	-	1052088	A	G	AE000513.1
-	-	1052234	.	C	AE000513.1
-	-	1052411	.	G	AE000513.1
1056019..1056357	hypothetical protein	1056348	.	C	AE000513.1
complement(1064215..1064925)	hydrolase, putative	1064290	.	G	AE000513.1
complement(1069192..1070088)	hypothetical protein	1069194	.	G	AE000513.1
complement(1069192..1070088)	hypothetical protein	1069708	G	.	AE000513.1

complement(1069192..1070088)	hypothetical protein	1069709	C	.	AE000513.1
complement(1069192..1070088)	hypothetical protein	1069710	G	.	AE000513.1
1071779..1072945	NADPH quinone oxidoreductase, putative	1071779	.	C	AE000513.1
1071779..1072945	NADPH quinone oxidoreductase, putative	1071829	.	C	AE000513.1
-	-	1073121	.	G	AE000513.1
complement(1075873..1076655)	hypothetical protein	1076215	G	.	AE000513.1
-	-	1077277	C	.	AE000513.1
1081696..1082061	hypothetical protein	1082021	.	C	AE000513.1
1082045..1082800	(3R)-hydroxymyristoyl-acyl carrier protei	1082259	C	.	AE000513.1
-	-	1100642	.	C	AE000513.1
-	-	1100740	.	C	AE000513.1
-	-	1106348	.	G	AE000513.1
1110055..1112328	ABC transporter, ATP-binding protein, EF-	1112028	.	G	AE000513.1
-	-	1112537	C	.	AE000513.1
1129614..1130732	prephenate dehydrogenase	1130547	C	G	AE000513.1

-	-	1134710	.	G	AE000513.1
1134718..1136772	single-stranded-DNA-specific exonuclease	1136609	.	G	AE000513.1
-	-	1145169	.	G	AE000513.1
complement(1147461..1148225)	hypothetical protein	1147504	.	C	AE000513.1
complement(1152328..1153038)	hypothetical protein	1152406	.	C	AE000513.1
1157470..1157997	conserved hypothetical protein	1157628	.	C	AE000513.1
1157470..1157997	conserved hypothetical protein	1157628	.	T	AE000513.1
1157470..1157997	conserved hypothetical protein	1157628	.	G	AE000513.1
-	-	1159254	G	.	AE000513.1
complement(1161195..1162103)	3-hydroxybutyryl-CoA dehydratase	1161635	A	G	AE000513.1
-	-	1168428	.	G	AE000513.1
-	-	1185927	.	C	AE000513.1
1205621..1205929	hypothetical protein	1205870	T	.	AE000513.1
1205621..1205929	hypothetical protein	1205871	C	G	AE000513.1
1205621..1205929	hypothetical protein	1205872	C	G	AE000513.1
1205621..1205929	hypothetical protein	1205873	C	A	AE000513.1

1205621..1205929	hypothetical protein	1205874	C	T	AE000513.1
1209784..1212849	hypothetical protein	1211737	A	G	AE000513.1
1219673..1220260	hypothetical protein	1220133	T	.	AE000513.1
1219673..1220260	hypothetical protein	1220187	C	.	AE000513.1
1232894..1233985	hypothetical protein	1233932	.	C	AE000513.1
1235582..1235986	hypothetical protein	1235591	G	.	AE000513.1
complement(1235983..1236942)	hypothetical protein	1236732	.	G	AE000513.1
complement(1245936..1247117)	homocitrate synthase	1247086	G	.	AE000513.1
1265444..1267246	DNA helicase, SNF2/RAD54 family	1265521	C	.	AE000513.1
1275795..1276199	hypothetical protein	1275805	.	C	AE000513.1
1275795..1276199	hypothetical protein	1275869	.	G	AE000513.1
-	-	1277438	.	G	AE000513.1
1291027..1291446	hypothetical protein	1291068	.	C	AE000513.1
complement(1292534..1293145)	conserved hypothetical protein	1292598	.	C	AE000513.1
complement(1307372..1308067)	hypothetical protein	1307999	.	G	AE000513.1

complement(1307372..1308067)	hypothetical protein	1308020	.	G	AE000513.1
complement(1307372..1308067)	hypothetical protein	1308021	T	C	AE000513.1
complement(1307372..1308067)	hypothetical protein	1308026	S	A	AE000513.1
complement(1307372..1308067)	hypothetical protein	1308028	.	G	AE000513.1
complement(1307372..1308067)	hypothetical protein	1308029	A	C	AE000513.1
complement(1307372..1308067)	hypothetical protein	1308030	G	A	AE000513.1
complement(1307372..1308067)	hypothetical protein	1308034	A	.	AE000513.1
-	-	1318403	.	G	AE000513.1
1323043..1323279	hypothetical protein	1323074	T	C	AE000513.1
1323043..1323279	hypothetical protein	1323075	G	T	AE000513.1
1323454..1324650	acyl-CoA dehydrogenase	1324318	C	G	AE000513.1
1323454..1324650	acyl-CoA dehydrogenase	1324319	G	C	AE000513.1
-	-	1327764	.	C	AE000513.1
1332428..1333861	multidrug-efflux transporter	1333766	.	G	AE000513.1

complement(1334225..1335019)	conserved hypothetical protein	1334807	A	.	AE000513.1
complement(1334225..1335019)	conserved hypothetical protein	1334811	.	C	AE000513.1
complement(1336292..1336690)	hypothetical protein	1336356	.	G	AE000513.1
complement(1336292..1336690)	hypothetical protein	1336376	C	.	AE000513.1
complement(1336760..1337806)	glycosyl hyrolase, family 3	1336760	T	C	AE000513.1
1342520..1344181	transport protein, putative	1342753	.	G	AE000513.1
1342520..1344181	transport protein, putative	1343009	.	G	AE000513.1
1350424..1351092	hydrolase	1350441	C	.	AE000513.1
1351086..1351622	hypothetical protein	1351524	.	C	AE000513.1
1363325..1364302	ABC transporter, ATP-binding protein	1363632	C	G	AE000513.1
1378605..1381670	DNA topoisomerase I	1378670	.	C	AE000513.1
complement(1406927..1407250)	hypothetical protein	1406938	.	G	AE000513.1

complement(1408946..1409383)	hypothetical protein	1409347	G	.	AE000513.1
-	-	1417882	.	G	AE000513.1
-	-	1417884	G	C	AE000513.1
complement(1418582..1421611)	penicillin-binding protein 1B McrB, putative	1419293	.	G	AE000513.1
-	-	1423853	A	G	AE000513.1
-	-	1443139	.	G	AE000513.1
complement(1451400..1452680)	hypothetical protein	1451624	.	C	AE000513.1
-	-	1456205	C	T	AE000513.1
-	-	1456209	C	T	AE000513.1
-	-	1456212	A	G	AE000513.1
-	-	1456216	A	G	AE000513.1
-	-	1456219	T	C	AE000513.1
-	-	1460634	A	.	AE000513.1
complement(1465188..1471010)	hypothetical protein	1465338	.	G	AE000513.1
complement(1465188..1471010)	hypothetical protein	1468015	G	C	AE000513.1
complement(1465188..1471010)	hypothetical protein	1470524	.	G	AE000513.1
complement(1470856..1475187)	hypothetical protein	1470933	.	C	AE000513.1

complement(1470856..1475187)	hypothetical protein	1474356	C	.	AE000513.1
-	-	1475208	.	G	AE000513.1
-	-	1475209	.	G	AE000513.1
-	-	1481060	.	C	AE000513.1
-	-	1481088	.	C	AE000513.1
complement(1498936..1499907)	lipase, putative	1499059	.	C	AE000513.1
complement(1498936..1499907)	lipase, putative	1499091	.	G	AE000513.1
complement(1498936..1499907)	lipase, putative	1499091	.	C	AE000513.1
-	-	1504300	.	G	AE000513.1
-	-	1521870	G	.	AE000513.1
-	-	1533823	.	C	AE000513.1
-	-	1538253	.	G	AE000513.1
1543319..1543720	hypothetical protein	1543632	.	G	AE000513.1
complement(1566493..1568223)	ftsE protein	1567364	.	G	AE000513.1
complement(1566493..1568223)	ftsE protein	1567364	.	C	AE000513.1
complement(1566493..1568223)	ftsE protein	1567366	G	C	AE000513.1
-	-	1572134	.	G	AE000513.1
-	-	1572569	.	C	AE000513.1
1577968..1578534	hypothetical protein	1578013	.	G	AE000513.1

complement(1584564..1585445)	hypothetical protein	1584708	C	.	AE000513.1
complement(1587670..1588800)	peptide ABC transporter, permease protein	1587965	A	C	AE000513.1
-	-	1593381	.	G	AE000513.1
complement(1605887..1606996)	glutamate racemase	1606957	.	C	AE000513.1
1621524..1622936	conserved hypothetical protein	1622844	.	C	AE000513.1
-	-	1628748	.	C	AE000513.1
-	-	1644529	C	.	AE000513.1
-	-	1644543	T	.	AE000513.1
-	-	1645961	.	G	AE000513.1
-	-	1651396	.	G	AE000513.1
-	-	1656768	A	G	AE000513.1
-	-	1656769	G	A	AE000513.1
complement(1660103..1661395)	hypothetical protein	1661111	.	G	AE000513.1
complement(1660103..1661395)	hypothetical protein	1661204	.	G	AE000513.1
complement(1660103..1661395)	hypothetical protein	1661207	.	C	AE000513.1
-	-	1661416	.	C	AE000513.1
-	-	1661493	.	G	AE000513.1
-	-	1664674	.	G	AE000513.1
1666372..1666692	hypothetical protein	1666629	.	C	AE000513.1

1666372..1666692	hypothetical protein	1666629	.	A	AE000513.1
1666372..1666692	hypothetical protein	1666629	.	G	AE000513.1
1668196..1668828	hypothetical protein	1668212	.	C	AE000513.1
-	-	1671409	.	C	AE000513.1
1680022..1681581	Mg(2+) chelatase family protein	1681486	T	G	AE000513.1
-	-	1720434	G	.	AE000513.1
-	-	1725535	G	.	AE000513.1
-	-	1776081	.	C	AE000513.1
1814915..1816354	mocR protein	1815401	.	G	AE000513.1
1814915..1816354	mocR protein	1815426	G	.	AE000513.1
-	-	1829905	.	G	AE000513.1
-	-	1829908	G	C	AE000513.1
-	-	1838361	.	G	AE000513.1
-	-	1840322	.	G	AE000513.1
complement(1883538..1883777)	hypothetical protein	1883541	C	.	AE000513.1
1887476..1888414	conserved hypothetical protein	1887543	C	.	AE000513.1
-	-	1888789	C	.	AE000513.1
1892037..1893227	chloromuconate cycloisomerase, putative	1892723	C	G	AE000513.1
1892037..1893227	chloromuconate	1892724	G	A	AE000513.1

	cycloisomerase, putative				
1892037..1893227	chloromuconate cycloisomerase, putative	1892727	G	C	AE000513.1
1906346..1906921	conserved hypothetical protein	1906884	.	G	AE000513.1
-	-	1928137	.	C	AE000513.1
-	-	1935000	.	G	AE000513.1
-	-	1935234	.	C	AE000513.1
1935271..1935453	hypothetical protein	1935418	.	G	AE000513.1
1940555..1941127	conserved hypothetical protein	1940620	C	.	AE000513.1
1942746..1945475	exonuclease SbcC	1943461	.	C	AE000513.1
1942746..1945475	exonuclease SbcC	1943472	.	G	AE000513.1
1942746..1945475	exonuclease SbcC	1943474	K	A	AE000513.1
1942746..1945475	exonuclease SbcC	1943484	T	G	AE000513.1
1942746..1945475	exonuclease SbcC	1943485	.	G	AE000513.1
1942746..1945475	exonuclease SbcC	1943531	T	C	AE000513.1
1942746..1945475	exonuclease SbcC	1944586	.	G	AE000513.1
1942746..1945475	exonuclease SbcC	1944594	.	G	AE000513.1
1942746..1945475	exonuclease SbcC	1944594	.	C	AE000513.1

1942746..1945475	exonuclease SbcC	1944809	A	G	AE000513.1
1942746..1945475	exonuclease SbcC	1944810	G	C	AE000513.1
1945630..1946460	hypothetical protein	1946367	.	C	AE000513.1
-	-	1946473	.	C	AE000513.1
complement(1952451..1953236)	conserved hypothetical protein	1952485	.	C	AE000513.1
complement(1963590..1964876)	3-oxoacyl-acyl carrier protein synthase II	1963745	G	C	AE000513.1
complement(1963590..1964876)	3-oxoacyl-acyl carrier protein synthase II	1963746	C	G	AE000513.1
1981234..1981764	ankyrin-related protein	1981601	.	G	AE000513.1
1989835..1990194	conserved hypothetical protein	1989862	C	.	AE000513.1
1994173..1996614	ATP-dependent protease LA	1995430	G	T	AE000513.1
1994173..1996614	ATP-dependent protease LA	1995441	C	G	AE000513.1
complement(2007783..2008421)	thymidine kinase	2007847	.	C	AE000513.1
complement(2007783..2008421)	thymidine kinase	2007847	.	G	AE000513.1

complement(2013610..2014671)	conserved hypothetical protein	2014654	.	C	AE000513.1
complement(2013610..2014671)	conserved hypothetical protein	2014654	.	G	AE000513.1
-	-	2018608	A	.	AE000513.1
complement(2019490..2020281)	conserved hypothetical protein	2020161	C	.	AE000513.1
-	-	2022950	.	G	AE000513.1
-	-	2031960	G	A	AE000513.1
-	-	2031964	G	C	AE000513.1
-	-	2031967	A	.	AE000513.1
-	-	2031968	G	C	AE000513.1
-	-	2031969	G	T	AE000513.1
-	-	2046695	.	G	AE000513.1
complement(2047650..2050013)	glutamine synthase	2048322	G	.	AE000513.1
complement(2047650..2050013)	glutamine synthase	2048325	.	G	AE000513.1
2050132..2050908	aminoglycoside N3`-acetyltransferase, type IV	2050457	T	C	AE000513.1
complement(2056384..2058351)	conserved hypothetical protein	2056841	.	G	AE000513.1
complement(2056384..2058351)	conserved hypothetical protein	2056841	.	C	AE000513.1
-	-	2068922	.	G	AE000513.1
-	-	2068940	.	G	AE000513.1

-	-	2069318	.	G	AE000513.1
-	-	2069323	.	C	AE000513.1
2072204..2073190	hypothetical protein	2073092	.	C	AE000513.1
2096369..2096737	hypothetical protein	2096659	C	.	AE000513.1
2101779..2103440	hypothetical protein	2101879	T	C	AE000513.1
2116835..2118277	molybdate metabolism regulator-related protein	2117392	A	G	AE000513.1
complement(2135694..2136146)	hypothetical protein	2135920	T	G	AE000513.1
complement(2136586..2138172)	conserved hypothetical protein	2137189	.	C	AE000513.1
complement(2144834..2145868)	conserved hypothetical protein	2144907	T	.	AE000513.1
complement(2144834..2145868)	conserved hypothetical protein	2144910	C	G	AE000513.1
2146441..2148099	ABC transporter, ATP-binding protein	2147407	.	C	AE000513.1
-	-	2148109	.	C	AE000513.1
-	-	2148895	.	G	AE000513.1
complement(2149081..2149761)	hypothetical protein	2149109	C	G	AE000513.1

complement(2149081..2149761)	hypothetical protein	2149110	.	C	AE000513.1
-	-	2150439	G	.	AE000513.1
-	-	2157569	.	G	AE000513.1
-	-	2157569	.	G	AE000513.1
2160561..2160971	hypothetical protein	2160661	G	C	AE000513.1
-	-	2164232	G	.	AE000513.1
-	-	2188924	G	.	AE000513.1
-	-	2188925	C	.	AE000513.1
2189795..2190067	hypothetical protein	2189942	G	A	AE000513.1
complement(2194404..2195789)	hypothetical protein	2194956	N	.	AE000513.1
complement(2194404..2195789)	hypothetical protein	2194958	.	G	AE000513.1
2202100..2203284	UDP-glucose 4-epimerase	2203078	.	G	AE000513.1
-	-	2215178	.	C	AE000513.1
-	-	2227283	C	.	AE000513.1
complement(2231724..2232062)	hypothetical protein	2231823	.	G	AE000513.1
-	-	2238344	.	C	AE000513.1
-	-	2238474	.	C	AE000513.1
2242761..2244233	methoxyneurosporene dehydrogenase	2244231	T	C	AE000513.1
-	-	2247372	G	.	AE000513.1

complement(2281403..2282122)	manganese ABC transporter, ATP-binding protein	2281720	C	.	AE000513.1
-	-	2314060	C	G	AE000513.1
complement(2339068..2340492)	birA bifunctional protein	2340149	.	G	AE000513.1
2346058..2347737	hypothetical protein	2347390	T	C	AE000513.1
2347866..2348678	hypothetical protein	2348609	.	C	AE000513.1
-	-	2365230	.	G	AE000513.1
complement(2365388..2367115)	glutathione-regulated potassium-efflux syste	2365390	.	G	AE000513.1
complement(2365388..2367115)	glutathione-regulated potassium-efflux syste	2365409	A	C	AE000513.1
complement(2401695..2402672)	conserved hypothetical protein	2402411	.	G	AE000513.1
2410637..2412484	DNA polymerase III, tau/gamma subunit	2412461	T	.	AE000513.1
-	-	2420014	A	.	AE000513.1
2421284..2422405	DNA-binding response regulator	2421928	.	G	AE000513.1
2421284..2422405	DNA-binding response regulator	2422010	.	C	AE000513.1

-	-	2431415	G	.	AE000513.1
-	-	2431599	C	.	AE000513.1
2433075..2434337	conserved hypothetical protein	2434200	C	.	AE000513.1
-	-	2435646	.	C	AE000513.1
-	-	2437558	C	.	AE000513.1
2445791..2446162	hypothetical protein	2446149	.	G	AE000513.1
-	-	2458891	G	.	AE000513.1
-	-	2459053	C	.	AE000513.1
2465337..2467286	acetyl-CoA synthase	2465575	G	.	AE000513.1
complement(2471534..2472199)	hypothetical protein	2471633	A	.	AE000513.1
-	-	2474213	.	G	AE000513.1
-	-	2474542	.	G	AE000513.1
2484833..2486380	ABC transporter, permease protein, CysTW family	2486190	T	C	AE000513.1
2486796..2488229	hypothetical protein	2486934	.	G	AE000513.1
2486796..2488229	hypothetical protein	2488045	T	C	AE000513.1
-	-	2490732	.	C	AE000513.1
complement(2508643..2511489)	hexagonally packed intermediate-layer surfac	2511220	G	A	AE000513.1
2512346..2513128	enoyl-CoA hydratase, putative	2513107	G	A	AE000513.1

complement(2514530..2515591)	conserved hypothetical protein	2515480	C	.	AE000513.1
-	-	2517684	.	C	AE000513.1
complement(2517685..2518812)	hypothetical protein	2518676	.	G	AE000513.1
-	-	2525930	.	C	AE000513.1
complement(2532961..2533398)	hypothetical protein	2533038	C	.	AE000513.1
-	-	2533406	.	G	AE000513.1
2533937..2534788	thiosulfate sulfurtransferase	2534551	T	C	AE000513.1
2537848..2538933	conserved hypothetical protein	2538821	.	C	AE000513.1
complement(2543403..2543936)	hypothetical protein	2543546	C	.	AE000513.1
complement(2547403..2549442)	conserved hypothetical protein	2548730	T	C	AE000513.1
2560973..2561443	hypothetical protein	2561376	.	G	AE000513.1
2567723..2568229	hypothetical protein	2567892	.	C	AE000513.1
-	-	2569064	G	C	AE000513.1
complement(2572383..2573195)	hypothetical protein	2573194	.	G	AE000513.1
-	-	2573225	.	G	AE000513.1
-	-	2573340	.	G	AE000513.1
2574755..2576245	hypothetical protein	2576197	.	C	AE000513.1

2576233..2576544	hypothetical protein	2576358	A	C	AE000513.1
-	-	2577432	G	C	AE000513.1
-	-	2577471	.	G	AE000513.1
-	-	2577471	.	C	AE000513.1
-	-	2577492	.	G	AE000513.1
complement(2590036..2590593)	conserved hypothetical protein	2590080	.	C	AE000513.1
-	-	2590637	.	C	AE000513.1
-	-	2609261	G	.	AE000513.1
-	-	2615343	A	C	AE000513.1
complement(2616672..2617097)	hypothetical protein	2616866	G	T	AE000513.1
-	-	2628828	.	C	AE000513.1
-	-	2644176	C	.	AE000513.1
-	-	19081	.	A	AE001825.1
-	-	26635	.	G	AE001825.1
-	-	27198	.	G	AE001825.1
complement(56537..56779)	hypothetical protein	56564	.	G	AE001825.1
complement(79518..80240)	hypothetical protein	79628	.	G	AE001825.1
91974..92366	hypothetical protein	91974	.	C	AE001825.1
91974..92366	hypothetical protein	92323	G	.	AE001825.1
-	-	95204	.	C	AE001825.1
97981..98715	hypothetical protein	98329	.	G	AE001825.1

97981..98715	hypothetical protein	98329	.	C	AE001825.1
102993..105422	hypothetical protein	103187	.	C	AE001825.1
complement(126441..127172)	hypothetical protein	126646	.	C	AE001825.1
complement(132390..133382)	acyl-CoA dehydrogenase, putative	132435	.	C	AE001825.1
complement(133289..134692)	hypothetical protein	133390	.	C	AE001825.1
161935..162603	phosphate transport system regulatory protei	162113	A	G	AE001825.1
complement(175372..176214)	thiamin-phosphate pyrophosphorylase	176167	G	.	AE001825.1
complement(176242..178065)	thiamin biosynthesis ThiC	177694	C	.	AE001825.1
184027..184851	conserved hypothetical protein	184065	.	G	AE001825.1
-	-	193733	.	G	AE001825.1
197036..198550	conserved hypothetical protein	197080	C	.	AE001825.1
-	-	220602	.	C	AE001825.1
-	-	222815	A	.	AE001825.1
-	-	225269	A	.	AE001825.1

227890..228774	conserved hypothetical protein	228597	.	G	AE001825.1
-	-	271780	C	.	AE001825.1
-	-	281718	.	C	AE001825.1
-	-	302667	G	C	AE001825.1
-	-	302894	T	C	AE001825.1
-	-	303032	.	C	AE001825.1
303497..303838	hypothetical protein	303753	.	G	AE001825.1
303829..305343	hypothetical protein	305178	C	G	AE001825.1
308594..309277	conserved hypothetical protein	309263	.	C	AE001825.1
308594..309277	conserved hypothetical protein	309263	.	G	AE001825.1
-	-	309515	.	C	AE001825.1
complement(310115..311401)	hypothetical protein	310201	C	.	AE001825.1
complement(310115..311401)	hypothetical protein	311205	.	G	AE001825.1
complement(310115..311401)	hypothetical protein	311242	A	.	AE001825.1
complement(310115..311401)	hypothetical protein	311301	T	.	AE001825.1
complement(310115..311401)	hypothetical protein	311317	C	.	AE001825.1

complement(310115..311401)	hypothetical protein	311324	A	.	AE001825.1
complement(310115..311401)	hypothetical protein	311346	C	.	AE001825.1
-	-	311411	C	.	AE001825.1
-	-	311438	A	.	AE001825.1
-	-	311441	C	.	AE001825.1
-	-	311452	A	.	AE001825.1
-	-	311456	C	.	AE001825.1
-	-	311468	C	.	AE001825.1
-	-	311491	C	.	AE001825.1
-	-	311544	C	.	AE001825.1
-	-	311578	C	.	AE001825.1
-	-	311593	C	.	AE001825.1
-	-	311599	C	.	AE001825.1
-	-	311616	A	.	AE001825.1
-	-	311632	C	.	AE001825.1
complement(311654..312718)	hypothetical protein	311674	C	.	AE001825.1
complement(311654..312718)	hypothetical protein	311684	C	.	AE001825.1
complement(311654..312718)	hypothetical protein	311685	A	.	AE001825.1
complement(311654..312718)	hypothetical protein	311713	C	.	AE001825.1
complement(311654..312718)	hypothetical protein	311839	C	.	AE001825.1

complement(311654..312718)	hypothetical protein	311853	C	.	AE001825.1
complement(311654..312718)	hypothetical protein	312058	G	.	AE001825.1
complement(311654..312718)	hypothetical protein	312144	.	C	AE001825.1
complement(311654..312718)	hypothetical protein	312219	T	.	AE001825.1
complement(311654..312718)	hypothetical protein	312283	C	.	AE001825.1
complement(311654..312718)	hypothetical protein	312293	C	.	AE001825.1
complement(311654..312718)	hypothetical protein	312421	C	.	AE001825.1
complement(311654..312718)	hypothetical protein	312431	C	.	AE001825.1
complement(311654..312718)	hypothetical protein	312446	C	.	AE001825.1
complement(311654..312718)	hypothetical protein	312449	C	.	AE001825.1
complement(311654..312718)	hypothetical protein	312572	C	.	AE001825.1
complement(311654..312718)	hypothetical protein	312595	C	.	AE001825.1

complement(311654..312718)	hypothetical protein	312711	.	G	AE001825.1
complement(312761..313165)	hypothetical protein	313057	A	.	AE001825.1
313191..315158	cell division protein FtsH	313915	C	.	AE001825.1
313191..315158	cell division protein FtsH	314602	C	.	AE001825.1
315247..316080	2,4-dihydroxyhept-2-ene-1,7-dioic acid aldolase	315487	C	.	AE001825.1
315247..316080	2,4-dihydroxyhept-2-ene-1,7-dioic acid aldolase	315624	G	.	AE001825.1
316139..316645	hypothetical protein	316621	.	G	AE001825.1
316696..319737	hypothetical protein	319681	.	C	AE001825.1
complement(320651..320992)	hypothetical protein	320768	.	C	AE001825.1
-	-	321029	.	G	AE001825.1
complement(321078..321617)	hypothetical protein	321170	.	G	AE001825.1
complement(323604..324353)	hypothetical protein	323973	G	C	AE001825.1
complement(329200..331329)	hypothetical protein	329439	.	G	AE001825.1

complement(329200..331329)	hypothetical protein	329439	.	C	AE001825.1
complement(331332..332087)	hypothetical protein	331334	.	G	AE001825.1
complement(340933..341400)	urease accessory protein UreE	341356	A	G	AE001825.1
-	-	345421	G	.	AE001825.1
356999..358186	hypothetical protein	358104	.	C	AE001825.1
390277..392547	methyl-accepting chemotaxis-related protein	392473	.	G	AE001825.1
390277..392547	methyl-accepting chemotaxis-related protein	392476	G	.	AE001825.1
-	-	395959	.	C	AE001825.1
404159..404749	hypothetical protein	404181	C	.	AE001825.1
-	-	26999	.	G	AE001826.1
41751..42122	hypothetical protein	42095	.	G	AE001826.1
44540..44977	hypothetical protein	44935	G	.	AE001826.1
44988..47798	hypothetical protein	46275	T	C	AE001826.1
44988..47798	hypothetical protein	46279	.	G	AE001826.1
44988..47798	hypothetical protein	46301	G	C	AE001826.1

44988..47798	hypothetical protein	46303	C	G	AE001826.1
44988..47798	hypothetical protein	46304	.	C	AE001826.1
44988..47798	hypothetical protein	46326	.	G	AE001826.1
82675..85335	hypothetical protein	84523	A	.	AE001826.1
82675..85335	hypothetical protein	85207	T	.	AE001826.1
82675..85335	hypothetical protein	85282	G	.	AE001826.1
85353..87587	serine protease, subtilase family	85360	G	.	AE001826.1
85353..87587	serine protease, subtilase family	85870	T	.	AE001826.1
85353..87587	serine protease, subtilase family	85874	G	.	AE001826.1
85353..87587	serine protease, subtilase family	85954	A	.	AE001826.1
85353..87587	serine protease, subtilase family	86049	A	.	AE001826.1
complement(87623..88486)	hypothetical protein	87910	C	.	AE001826.1
-	-	94328	.	C	AE001826.1
-	-	106291	C	.	AE001826.1

157173..162293	RNA helicase, putative	158665	C	.	AE001826.1
complement(176244..177437)	hypothetical protein	176400	.	G	AE001826.1
73..384	cytochrome P450-related protein	145	.	C	AE001827.1
-	-	594	.	C	AE001827.1
-	-	596	T	C	AE001827.1
-	-	3119	.	G	AE001827.1
-	-	3595	C	T	AE001827.1
-	-	8096	.	G	AE001827.1
complement(8308..10218)	hypothetical protein	9122	G	C	AE001827.1
-	-	12161	.	G	AE001827.1
-	-	12480	.	C	AE001827.1
-	-	12483	.	C	AE001827.1
-	-	12499	C	T	AE001827.1
13519..14157	N-acetylmuramoyl-L-alanine amidase, putative	14100	.	C	AE001827.1
-	-	14158	A	.	AE001827.1
-	-	14189	T	.	AE001827.1
-	-	14190	N	.	AE001827.1
complement(16040..16852)	hypothetical protein	16189	.	C	AE001827.1
-	-	17763	.	C	AE001827.1
complement(20836..21270)	transposase-related	20873	C	.	AE001827.1

complement(20836..21270)	transposase-related	20956	C	.	AE001827.1
complement(20836..21270)	transposase-related	21063	G	.	AE001827.1
complement(20836..21270)	transposase-related	21219	G	.	AE001827.1
-	-	21786	A	.	AE001827.1
-	-	21821	T	.	AE001827.1
-	-	21847	T	.	AE001827.1
-	-	21857	G	.	AE001827.1
-	-	22040	G	.	AE001827.1
-	-	22069	.	C	AE001827.1
-	-	22136	.	C	AE001827.1
-	-	22195	C	.	AE001827.1
-	-	22310	C	.	AE001827.1
-	-	22613	T	.	AE001827.1
-	-	22706	G	.	AE001827.1
complement(22901..23269)	hypothetical protein	23006	.	C	AE001827.1
complement(22901..23269)	hypothetical protein	23187	C	.	AE001827.1
complement(23296..23892)	hypothetical protein	23302	.	G	AE001827.1
complement(23296..23892)	hypothetical protein	23390	T	.	AE001827.1
complement(23296..23892)	hypothetical protein	23524	.	A	AE001827.1

complement(23296..23892)	hypothetical protein	23528	A	.	AE001827.1
complement(23296..23892)	hypothetical protein	23550	T	.	AE001827.1
-	-	23971	G	.	AE001827.1
-	-	24014	G	.	AE001827.1
-	-	24036	C	.	AE001827.1
-	-	24157	T	.	AE001827.1
-	-	24162	.	C	AE001827.1
-	-	24305	G	.	AE001827.1
-	-	24390	C	.	AE001827.1
-	-	24400	C	.	AE001827.1
-	-	24492	G	.	AE001827.1
-	-	24502	T	.	AE001827.1
-	-	24715	G	T	AE001827.1
-	-	24716	T	G	AE001827.1
-	-	24874	.	A	AE001827.1
-	-	24903	.	C	AE001827.1
24950..25609	hypothetical protein	25499	T	.	AE001827.1
-	-	25711	.	C	AE001827.1
27711..28106	hypothetical protein	27911	.	C	AE001827.1
30272..31138	transposase, putative	30415	G	T	AE001827.1
30272..31138	transposase, putative	30418	C	G	AE001827.1
30272..31138	transposase, putative	30442	T	C	AE001827.1
30272..31138	transposase, putative	30451	T	C	AE001827.1

30272..31138	transposase, putative	30457	C	G	AE001827.1
30272..31138	transposase, putative	30463	T	C	AE001827.1
30272..31138	transposase, putative	30466	A	G	AE001827.1
30272..31138	transposase, putative	30487	C	G	AE001827.1
30272..31138	transposase, putative	30535	G	T	AE001827.1
30272..31138	transposase, putative	30541	C	T	AE001827.1
30272..31138	transposase, putative	30547	C	T	AE001827.1
30272..31138	transposase, putative	30550	G	A	AE001827.1
30272..31138	transposase, putative	30568	T	C	AE001827.1
30272..31138	transposase, putative	30587	A	C	AE001827.1
30272..31138	transposase, putative	30592	A	G	AE001827.1
30272..31138	transposase, putative	30595	T	C	AE001827.1
30272..31138	transposase, putative	30598	T	C	AE001827.1
30272..31138	transposase, putative	30619	A	G	AE001827.1
30272..31138	transposase, putative	30631	G	T	AE001827.1
30272..31138	transposase, putative	30673	C	T	AE001827.1
30272..31138	transposase, putative	30679	T	C	AE001827.1

30272..31138	transposase, putative	30681	G	A	AE001827.1
30272..31138	transposase, putative	30724	A	G	AE001827.1
30272..31138	transposase, putative	30749	T	G	AE001827.1
30272..31138	transposase, putative	30750	C	T	AE001827.1
30272..31138	transposase, putative	30789	G	A	AE001827.1
30272..31138	transposase, putative	30799	A	G	AE001827.1
30272..31138	transposase, putative	30814	T	A	AE001827.1
30272..31138	transposase, putative	30832	T	C	AE001827.1
30272..31138	transposase, putative	30838	A	G	AE001827.1
30272..31138	transposase, putative	30850	T	C	AE001827.1
30272..31138	transposase, putative	30854	T	C	AE001827.1
30272..31138	transposase, putative	30856	A	G	AE001827.1
30272..31138	transposase, putative	30862	A	G	AE001827.1
30272..31138	transposase, putative	30865	C	T	AE001827.1
30272..31138	transposase, putative	30904	A	C	AE001827.1
30272..31138	transposase, putative	30913	A	G	AE001827.1
30272..31138	transposase, putative	30925	G	A	AE001827.1

30272..31138	transposase, putative	30932	T	C	AE001827.1
30272..31138	transposase, putative	30937	C	A	AE001827.1
30272..31138	transposase, putative	30940	G	T	AE001827.1
30272..31138	transposase, putative	30952	G	C	AE001827.1
30272..31138	transposase, putative	30962	T	C	AE001827.1
30272..31138	transposase, putative	30988	T	C	AE001827.1
30272..31138	transposase, putative	30991	G	C	AE001827.1
30272..31138	transposase, putative	31010	C	A	AE001827.1
30272..31138	transposase, putative	31075	A	G	AE001827.1
30272..31138	transposase, putative	31087	A	T	AE001827.1
30272..31138	transposase, putative	31091	C	G	AE001827.1
-	-	33781	.	C	AE001827.1
-	-	34155	.	C	AE001827.1
-	-	35076	.	G	AE001827.1
-	-	42608	.	C	AE001827.1
